# Coordinating perspectives: *De se* and taste attitudes in communication

**DOI:** 10.1080/0020174X.2019.1612773

**Published:** 2019-05-12

**Authors:** Dirk Kindermann

**Affiliations:** Institut für Philosophie, Universität Wien, Wien, Austria

**Keywords:** De se attitudes, predicates of personal taste, assertion, self-location, centered worlds, multicentered worlds

## Abstract

The received picture of linguistic communication understands communication as the transmission of information from speaker's head to hearer's head. This picture is in conflict with the attractive Lewisian view of belief as self-location, which is motivated by *de se* attitudes – first-personal attitudes about oneself – as well as attitudes about subjective matters such as personal taste. In this paper, I provide a solution to the conflict that reconciles these views. I argue for an account of mental attitudes and communication on which mental content and speech act content is understood as sets of *multicentered worlds* – roughly, possible worlds ‘centered’ on a sequence of individuals at a time. I develop a Stalnakerian model of communication based on multicentered worlds content, and I provide a suitable semantics for personal pronouns and predicates of personal taste. The resulting picture is one on which the point of conversation is the coordination of individual perspectives.

## 1. Introduction

There is, or appears to be, a conflict between, on the one hand, the perspectival nature of many of our attitudes and, on the other hand, the received picture of linguistic communication. According to this picture, there is a single content which the speaker believes, expresses in speech, and which hearers come to believe if they understand and trust the speaker. But it is difficult to see how this picture fits with a popular account of two kinds of perspectival attitudes: so-called *de se* attitudes, i.e. first-personal attitudes about oneself, such as the belief *that I am hungry*; and ‘subjective’ attitudes about matters such as personal taste, like the belief *that liquorice is tasty*. According to this account, mental attitudes have so-called *centered content* – roughly, content whose truth depends on an individual at a world and time. I will argue that the conflict between the received picture of communication and the centered content view of perspectival attitudes can be resolved without giving up either of these attractive views. The solution I will propose is a unified account of mental attitudes and linguistic communication on which content is modeled not in terms of centered worlds but as a set of *multicentered worlds* – roughly, possible worlds that are ‘centered’ on a sequence of individuals at a time.

It has long been argued that *de se* attitudes – first-personal attitudes about oneself – motivate abandoning a simple view of mental attitudes according to which they are relations between a subject and a proposition (a set of possible worlds or a structured proposition that is true or false *simpliciter*).^1^ On Lewis's influential proposal, the content of a thought one would express by using the words ‘I am hungry’ is a set of centered worlds, where a centered world is a possible world ‘centered’ on an individual at a time. To believe *that I am hungry* is to locate oneself in the set of centered worlds whose center is hungry. A growing number of philosophers and linguists also argue that thoughts about ‘subjective’ matters such as personal taste have centered content.^2^ To believe that liquorice is tasty is to locate oneself in the set of centered worlds to whose center liquorice tastes good.

How do we communicate these self-locating beliefs? The standard picture of communication says, very roughly, that we exchange information by simply passing it on, from speaker's head to hearer's head. But this widely endorsed picture is in conflict with self-locating belief. If I believe that I am hungry, and I say to you, ‘I am hungry’, you do *not* come to believe the content I believe. For that would be for you to believe that you are hungry. Instead you come to believe a content about me, namely that I am hungry.

The conflict at hand suggests that we reject either the natural standard picture of communication or the elegant Lewisian account of self-locating belief. I will argue that neither is necessary. I will begin by stating the self-locating account of belief and other attitudes (Section 2), the received picture of linguistic communication (Section 3), and the conflict between these two views (Section 4). Then I will show that the multicentered worlds view affords an attractive reconciliation of the two views, and I will develop the multicentered worlds view in a broadly Stalnakerian picture of communication (Sections 5–11). Along the way, I will propose a suitable semantics for pronouns (‘I’, ‘you’) and predicates of personal taste (‘tasty’, ‘fun’) that delivers multicentered worlds content (Sections 7 and 8), and I will discuss the pragmatics of discourse about matters of taste (Sections 9–11).

## 2. Centered content

*De se* attitudes are thoughts about oneself when one thinks about oneself in the first-person way. They are thoughts one would typically express with a sentence containing a 1st-personal pronoun (‘I’, ‘me’, ‘my’).^3^ David Lewis famously argued that the objects of *de se* attitudes cannot be understood as possible worlds propositions. Rather, he suggested, their contents must be (or determine) sets of *centered worlds*. A *centered world* is a possible world ‘centered’ on an individual inhabiting the world at a time. Just like possible worlds can be understood as ways the world might be, centered worlds can be understood as ways one might be in the world, as possible locations in logical space, or as perspectives one might have on the world. A centered world can be represented by an ordered triple ⟨w,t,x⟩ consisting of a world *w*, a time *t*, and an individual *x* inhabiting *w* at *t*.^4^ The content of my belief *that I am hungry* is the set of centered worlds in which the center is hungry:

(1) *HUNGRY*: {⟨w,t,x⟩: *x* is hungry in *w* at *t*}.

A number of arguments have been proffered which suggest that *de se* attitudes cannot adequately be described by a simple view of mental attitudes on which they are relations between a subject and a proposition (possible worlds proposition or structured proposition true or false *simpliciter*). Let me quickly rehearse what I consider the strongest of these arguments: similarity arguments (to borrow Egan's (2010b) term).^5^

Mad Heimson believes that he is Hume, a belief he would express by saying ‘I am Hume.’ Hume, of course, also believed of himself that he is Hume. Hume and Heimson share a belief, they are doxastically similar, which explains similarities in their actions (given that their desires and background beliefs are similar). They introduce themselves as ‘David Hume’, get angry when they hear Hume being badmouthed, sign with ‘David Hume’, and so on. But there is no relevant possible worlds proposition that both Heimson and Hume believe that would explain this doxastic similarity. Why not? Heimson and Hume are worldmates. So any candidate possible worlds proposition is either true at their world or false at their world. If it is true, then both Heimson and Hume have a true belief. If it is false, both Heimson and Hume have a false belief. But Hume is right in believing that he is Hume because he *is* Hume, and Heimson is wrong in so believing. So the shared object of their beliefs, which explains their similarities in action, cannot be a possible worlds proposition. Lewis concludes that the shared object of Heimson's and Hume's beliefs is the property *being Hume*, which each of them self-attributes.^6^ Equivalently, we can say that the shared object of their beliefs is the centered content that is the set of centered worlds whose center is Hume.

On a standard possible worlds account, one's overall belief state determines a set of possible worlds, the possible worlds compatible with what one believes. Analogously, on the centered worlds account, one's overall belief state determines a set of centered worlds, the set of centered worlds compatible with what one believes. A centered world ⟨w,t,x⟩ is compatible with what one believes iff one's beliefs do not rule out the possibility that one is *x* in *w* at *t*. One believes a centered worlds content *p* iff every centered world compatible with what one believes is contained in *p*. Hume believes that he is Hume iff every centered world compatible with what he believes is contained in

(2) *HUME*: {⟨w,t,x⟩: *x* is Hume in *w* at *t*}.

His belief in *HUME* at some time *t_1_* is true iff in addition, his actual location at *t_1_*, $\langle @,\;t_{1},\;$Hume⟩, is a member of *HUME*.

Lewis speaks of belief as self-attribution of properties. On centered worlds talk, belief is self-location in a set of centered worlds. Since properties correspond to sets of centered worlds, we will switch back and forth between these equivalent ways of talking.^7^

On the centered worlds account, *all* attitudes have centered content.^8^ However, not all centered contents are *de se* contents. Following Egan's (2006, 107) terminology, we can call a centered content *p boring* if it does not distinguish between locations in a world. More precisely, *p* is boring iff for every world *w* and pairs $\left\langle t_{1},\;x\right\rangle$, $\left\langle t_{2},\;y\right\rangle$ of individuals inhabiting *w* at times $t_{1}$ and $t_{2}$, respectively, *p* contains ⟨*w*, *t*_1_, *x*⟩ iff it contains $\left\langle w,\;t_{2},\;y\right\rangle$. Because boring centered contents distinguish between worlds but not between locations in a world, they are equivalent to possible worlds contents.^9^ *De se* contents do distinguish between locations in a world. They are *interesting*. A centered content *p* is interesting iff there is a world *w* and pairs $\left\langle t_{1},\;x\right\rangle$, $\left\langle t_{2},\;y\right\rangle$ of individuals inhabiting *w* at times $t_{1}$ and $t_{2}$, respectively, such that *p* contains $\left\langle w,\;t_{1},\;x\right\rangle$ but not $\left\langle w,\;t_{2},\;y\right\rangle$.

Egan (2005, 2007, 2010a), Lasersohn (2005) and Stephenson (2007a) argue that the contents of our attitudes towards ‘subjective’ matters like personal taste are best understood as interesting centered contents as well. Intuitively, the truth of claims about what is tasty, fun, or entertaining depends not just on what the objects concerned are like, but on some subject not made explicit. In most judgments of taste, this subject is the thinker, or speaker, herself. Note, for instance, the bizarreness of uttering, or thinking, ‘This cookie is tasty, but I hate how it tastes.’ On the centered worlds account, to believe that a particular cookie is tasty is to locate oneself in the set of centered worlds to whose center the cookie tastes good:

(3) *COOKIE*: {⟨w,t,x⟩: the (contextually salient) cookie tastes good to *x* in *w* at *t*}.

*COOKIE* is an interesting centered content. It can be true of one person and false of another. If the cookie happens to taste good to Ben, he is right in believing *COOKIE*, while Anna is wrong in believing *COOKIE* if the cookie does not taste good to her. Ben can correctly judge it true that the cookie is tasty, and Anna can correctly judge it false that the same cookie is tasty.

Let me offer another traditional motivation for the interestingness of taste contents.^10^ If Ben says, ‘This cookie is tasty’, and Anna replies, ‘No/I disagree/You're wrong. It's not tasty’, it is natural to regard them as disagreeing, even contradicting each other. (Note the felicity of the disagreement makers ‘No’, ‘I disagree’, and ‘You're wrong.’) Their disagreement is easily explained if Ben is asserting *COOKIE* and Anna is asserting the negation of *COOKIE*. The two contents contradict each other; there is no centered world at which *COOKIE* and its negation are both true. The main alternative view, on which Ben and Anna express boring contents – simple indexical contextualism – has trouble explaining the disagreement by appeal to the expressed contents. On the simple contextualist view, Ben and Anna each make claims about their own taste:

(4) Ben: {⟨w,t,x⟩: the cookie tastes good to Ben in *w* at *t*}
(5) Anna: {⟨w,t,x⟩: the cookie does not taste good to Anna in *w* at *t*}

(4) and (5) are compatible – it is possible for the cookie to taste good to Ben but not to Anna at the same world and time – so the disagreement cannot be due to contradictory contents.^11^

There are important differences between *de se* beliefs and beliefs about matters of personal taste. For the moment, however, notice their similarities. Crucially, both kinds of belief are beliefs in *interesting* centered contents and follow an egocentric belief norm:

**Table:** 

Egocentricity	Believe *p* only if *you yourself* are correctly located by *p*.

Importantly, Egocentricity requires only the believer's correct location to be contained in *p*. An agent's *de se* belief that she is hungry is correct as long as that agent is hungry, even if she were to be the only person in logical space ever to be hungry. Similarly, an agent appropriately believes that some cookie is tasty as long as she herself is such that the cookie tastes good to her, even if she were the only person ever to enjoy its taste.^12^

In addition to the interestingness of content and the egocentricity of belief, *de se* attitudes and attitudes about taste also have in common an account of believing-alike in terms of shared content. When Ben and Anna each believe, *I am hungry*, their similar disposition to act is explained by the shared interesting centered content of their beliefs. Likewise, when they each believe, *This wine is tasty*, they are disposed to similar behavior (given similar background beliefs and desires); they are reaching for their glass frequently, will not refuse a refill, etc. Their similar disposition can also be explained by their believing-alike, which is accounted for by the shared content: {⟨*w*,*t*,*x*⟩: the (contextually salient) wine tastes good to *x* in *w* at *t*}.

As it stands, the self-location account of *de se* and subjective attitudes is in conflict with the standard picture of linguistic communication. In the next two sections, I first introduce the picture and then present the conflict.

## 3. The Lockean picture of communication

Received wisdom paints a simple and attractive picture of linguistic communication as the transfer of information. This picture is famously expressed by Locke (1690):

They suppose their words to be marks of the ideas in the minds also of other men, with whom they communicate: for else they should talk in vain, and could not be understood, if the sounds they applied to one idea were such as by the hearer were applied to another, which is to speak two languages. But in this men [ … ] think it enough that they use the word, as they imagine, in the common acceptation of that language; in which they suppose that the idea they make it a sign of is precisely the same to which the understanding men of that country apply that name. (*An Essay Concerning Human Understanding*, book III, ch. 2, §4)

The picture attributed to Locke and arguably endorsed by much of twentieth century philosophy is this: a speaker succeeds in communicating when she has an idea in her head and uses the words which express this idea in language and which arouse in the hearer the very same idea. The speaker's mental content is, as it were, transported from her head to the hearer's head, who comes to share this content.^13^

On the Lockean picture, one kind of content plays the following three roles:

1. Speaker's mental content: what the speaker believes and intends to communicate
2. Speech act content: what the speaker's (assertoric) speech act literally expresses
3. Hearer's mental content: what the hearer comes to believe, if she understands and trusts the speaker

If I believe that snow is white and intend to tell you, and I assert the right words, ‘Snow is white’, I express my very belief. If you understand what I assert and trust me, you come to have a belief with the same content as I.

A theory of communication needs to say what plays these three roles. It also needs to say how Speaker's mental content, Speech act content, and Hearer's mental content are related:

Belief-speech coordination: the connection between the speaker's belief content and the content of the speech act
Speech-belief coordination: the connection between the speech act's content and the hearer's belief content

The Lockean picture again offers a straightforward account: These connections are simply the identity relation. Note that Speech act content is what the expressions used in a speech act semantically express. It coincides, roughly, with Grice's notion of *what the speaker says* rather than with Grice's *what the speaker means*, which may involve pragmatically implicated content.^14^

## 4. The conflict

There is *prima facie* evidence that we do communicate self-locating information. If mad Heimson were to ask a sympathetic contemporary, ‘Who am I?’ and received the answer, ‘You are Heimson’, it seems that he would be told just the piece of *de se* information that could set him straight.^15^ But this datum is in conflict with the Lockean picture. Suppose Ben has the *de se* belief that he is hungry and says to Anna, ‘I am hungry.’ The Lockean picture predicts that he is expressing the interesting centered content of his belief, which Anna will come to believe if she understands and accepts his assertion. That is, Anna will come to locate *herself* in the content and will thus believe *de se* that *she* is hungry. But what Ben communicates is obviously some other information – information Anna grasps if she comes to have a belief to the effect that the speaker, Ben, is hungry. Call this problem the *de se problem*.^16^

The problem for self-locating belief on the Lockean picture gets worse. It may seem that the right conclusion to draw from the *de se* problem is that hearers systematically *infer* an appropriate self-locating belief centered on themselves from the fact that the speaker asserted a content centered on herself. For instance, Anna may infer the centered content such that the center is being addressed by someone hungry from the fact that Ben asserted the centered content such that the center is hungry. This is, roughly, the conclusion that ‘recentering’ accounts of *de se* communication draw (see e.g. Heim 2004; Weber 2013). But inferring a self-locating belief in this way is not what happens in the communication of beliefs about matters of taste. Suppose Ben believes that some cookie is tasty and says to Anna, ‘This cookie is tasty’, thereby expressing the centered content *COOKIE*.

(3) *COOKIE*: {⟨w,t,x⟩: the (contextually salient) cookie tastes good to *x* in *w* at *t*}.

What will Anna come to believe if she understands and trusts him? She will *not* just come to locate herself in a content such that the center is addressed by someone to whom the cookie tastes good. On the contrary, if Anna understands and accepts the claim, she will come to have the very self-locating belief that Ben has, viz. a belief with the centered content such that the cookie tastes good to the center. For Anna to accept an assertion of ‘This cookie is tasty’ is for her to locate *herself* in a cookie-liking location.

Centered worlds *de se* content and centered worlds content about matters of taste play incompatible roles in communication. With successful assertions about taste, the hearer comes to believe the same centered content as the speaker. With successful assertions about oneself, the hearer does not come to believe the same centered content. Call this problem the *incompatibility problem*.

It may seem that we have to give up either the centered content belief model or the Lockean picture of communication. But this would be hasty. In the next sections, I will propose an account that preserves the simplicity of the Lockean picture and the self-locating nature of belief by modifying the notion of centered content.^17^

## 5. Multicentered worlds

A centered world is a possible way one individual may be. Centered worlds suffice for the modeling of belief as *self*-location, but not for communication. In communication, we are not just trying to locate ourselves individually. We are trying to locate ourselves as a group. We are trying to arrive at a common view about our collective location and everyone's position in it. And for that, the possible ways different individuals may be need to be represented. If I tell you, ‘It's my turn’, I am talking about myself in terms of my own possibilities. If I tell you, ‘It's your turn’, I am talking about you in terms of your possibilities. The fundamental problem with centered worlds content on the Lockean picture of communication is that the single center needs to sometimes represent the speaker, sometimes the addressee, and sometimes both.

The problem can be solved by introducing a *sequence* of centers. A *multicentered world*, or *sequenced world*, is a possible world centered on a number of individuals at a time.^18^ It is a possible way that a plurality of individuals might be that does not conflate their individual possibilities. Formally, a multicentered world is a triple consisting of a world *w*, a time *t*, and a sequence of individuals $\left\langle x_{1},\;\ldots ,\;x_{n}\right\rangle$ inhabiting *w* at *t*. A multicentered worlds content *p* is the set of $\left\langle w,\;t,\;\langle x_{1},\;\ldots ,\;x_{n}\rangle \right\rangle$-triples such that *p* is true at $\left\langle w,\;t,\;\langle x_{1},\;\ldots ,\;x_{n}\rangle \right\rangle$. Lewis thought of centered worlds contents as properties of individuals. Similarly, multicentered worlds contents can be thought of as properties of ordered *n*-tuples of individuals.^19^

I will here present a multicentered worlds model of communication that is a based on Ninan's (2010b) and Torre's (2010) accounts, which use multicentered worlds content to provide speech act content for *de se* thoughts. What I will show is that a suitably developed multicentered worlds model provides a solution to the conflict that preserves the Lockean picture of communication, for both *de se* and subjective attitudes. It yields a unified account of belief and communication for *de se* contents as well as contents about matters of taste.^20^

Speaker's mental content, Speech act content, and Hearer's mental content are now sets of multicentered worlds, with one slot in the sequence for each conversational participant. Whose possibilities each slot carves out must be stable in communication. Otherwise our two problems would persist. If the first center, say, were to carve out Ben's possibilities when he believes the content, but were to carve out Anna's possibilities when she comes to believe the content, their individual possibilities would again be conflated. In order to stabilize what the content of speech acts and beliefs held during conversation represents, we relativize it to a *conversational context c* = $\left\langle w_{c},\;t_{c},\;\langle x_{1},\;\ldots ,\;x_{n}\rangle \right\rangle$ – a triple of a world $w_{c}$, time $t_{c}$, and an ordered list of conversational participants $\left\langle x_{1},\;\ldots ,\;x_{n}\right\rangle$ in $w_{c}$ at $t_{c}$. Call the ordered list of conversational participants the *conversational sequence*. Who the participants to a conversation are depends on the mutually recognized intentions of speaker and audience. The order of the participants in the conversational sequence does not matter, as long as we keep it stable for the entire conversation. That is, for the purposes of modeling a conversation by a succession of conversational contexts, we have to pick the same conversational sequence for each of them.^21^

Let us see how this helps with *de se* communication. We stipulate that for the conversation between Ben and Anna in $w_{c}$ at $t_{c}$, the conversational context is $\langle w_{c},\;t_{c},\;\langle$Ben, Anna⟩⟩. Then the content of Ben's assertion of ‘I am hungry’ is the multicentered worlds content *HUNGRY_1_*:

(6) *HUNGRY_1_*: {$\left\langle w,\;t,\;\langle x_{1},\;x_{2}\rangle \right\rangle$: $x_{1}$ is hungry in *w* at *t*}

*HUNGRY_1_* says, roughly, that the first center $x_{1}$ is hungry. Given the conversational sequence ⟨Ben, Anna⟩, Ben's possibilities are represented by the first center, and Anna's possibilities by the second center. So for Ben to believe *HUNGRY_1_* is for him to believe *de se* that he is hungry. For Anna to believe *HUNGRY_1_* is for her to believe *de te* – ‘of you’ – that Ben is hungry. It is not for her to believe *de se* that she herself is hungry. So if Ben believes what he says and if Anna understands and accepts Ben's assertion, he and Anna will come to believe the same multicentered worlds content *HUNGRY_1_*. However, their doxastic states are not exactly the same, as they dispose them to different actions. (We will come back to this difference in Section 6.) This solves the *de se* problem.

Talk about taste need not distinguish between centers in the same way that *de se* communication must. If Ben successfully communicates ‘This cookie is tasty’ to Anna, they will each come to locate themselves in a cookie-liking location. On the multicentered world picture, we get this result if taste contents place conditions on every center. Let us again take $\langle w_{c},\;t_{c},\;\langle$Ben, Anna⟩⟩ as the conversational context. If Ben believes and asserts ‘This cookie is tasty’ in *c*, he expresses the multicentered worlds content *COOKIE*_1&2_:

(7) *COOKIE*_1&2_: {$\left\langle w,\;t,\;\langle x_{1},\;x_{2}\rangle \right\rangle$: the cookie tastes good to $\left\langle x_{1},\;x_{2}\right\rangle$ in *w* at *t*}

If communication is successful and Anna accepts Ben's assertion relative to the conversational sequence ⟨Ben, Anna⟩, she comes to locate herself in the set of multicentered worlds such that the cookie tastes good to *all* centers. And that seems right. If Ben wishes to establish that the cookie is tasty by asserting ‘This cookie is tasty’, he has succeeded if they both locate themselves among the cookie-likers. This solves the incompatibility problem: *de se* and subjective multicentered worlds contents do not play incompatible roles in communication. The communication of either is successful in case the hearer comes to believe the same content the speaker believed and expressed in speech.

Belief in *COOKIE*_1&2_ is different from purely egocentric belief whose correctness depends only on one's own correct location. Anna should believe *COOKIE*_1&2_ only if she believes that the cookie tastes good to the speaker and that it tastes good to herself. The latter belief egocentrically concerns her taste, the former is safe as long as she takes Ben's assertion to be sincere. Multicentered worlds content on the Lockean picture captures the fact that success in the communication of subjective, evaluative claims involves acceptance of a common perspective on the matter. In contrast, suppose ‘This cookie is tasty’ expressed the interesting *de se* content *COOKIE_1_*:

(8) *COOKIE*_1_: {$\left\langle w,\;t,\;\langle x_{1},\;x_{2}\rangle \right\rangle$: the cookie tastes good to $x_{1}$ in *w* at *t*}

Anna could felicitously accept *COOKIE_1_* by saying ‘Yes, that's right’ if she has reason to believe that the cookie tastes good to the speaker. But it seems that in her reply she takes on a different commitment: that she agrees on the taste of the cookie. Thus, bare taste claims need to express contents that interestingly locate every participant to the conversation (*COOKIE_1&2_*), if we want to capture their communicational role and ensure the right predictions for agreement and disagreement.

This is, in a nutshell, the multicentered worlds solution to the conflict between the Lockean picture of communication and a self-location account of belief and other attitudes. In the rest of the paper, I will develop the multicentered worlds view in more detail. I will first talk about the notion of belief (Section 6), will then address semantic questions (Sections 7–8), and will finally turn to the pragmatics of discourse about oneself and about matters of taste (Sections 9–11).

## 6. Belief in context

Multicentered worlds content, on the Lockean picture, is what is expressed and believed by speaker and audience. To believe a multicentered worlds content in a conversational context is to have a belief with a content whose multicentered worlds have sequences with as many individuals as there are parties to the conversation. It is to locate oneself as well as everyone else in the conversation; it is to locate the group of which one is a member, in a way that allows for the perspectives of the members to differ. Belief in centered worlds content is self-location. Belief in multicentered worlds content in conversation is location of the conversational group of which one is a member; it is self-and-group-location.^22^

The notion of believing a multicentered worlds content must be relativized to a conversational context and a believer. Here is why. Suppose Lingens says to his cousin Ortcutt, ‘I am tired of reading.’ If he is communicating successfully, then relative to the conversational context $\langle w_{c},\;t_{c},\;\langle$Lingens, Ortcutt⟩⟩ they will both end up believing *TIRED_1_*:

(9) *TIRED_1_*: {$\left\langle w,\;t,\;\langle x_{1},\;x_{2}\rangle \right\rangle$: $x_{1}$ is tired of reading in *w* at *t*}

But even when Lingens and Ortcutt believe the same multicentered worlds content *TIRED_1_* relative to the conversational context, there is still an important difference between their belief states. Their beliefs will dispose them to different actions – perhaps a disposition to stop reading for Lingens, and perhaps a disposition to say, ‘Why don't you take a break?’ for Ortcutt. This difference in belief states is accounted for by relativizing belief to agents in conversational contexts. We will say that in a conversational context $\langle w_{c},\;t_{c},\;\langle$Lingens, Ortcutt⟩⟩, Lingens 1-believes *TIRED_1_* (he comes first in the conversational sequence) and Ortcutt 2-believes *TIRED_1_*. Below is the definition of n-Belief in a conversational context. Somewhat informally, it says that an agent *n*-believes a multicentered worlds content *p* in a conversational context just in case (i) the agent is the *n*th member of the conversational sequence, (ii) the agent uniquely stands in relations to every member of the conversational sequence which establish a conversation between them (from which it follows that the conversational roles of speaker, hearer, and (intended 2nd-person) addressee(s) are occupied^23^), and (iii) the agent believes that she might be the *n*th member of a group of which *p* is true. The full account of multicentered worlds belief is as follows:

n-Belief in a conversational context

An agent *A* *n*-believes a multicentered worlds content {$\left\langle w,\;t,\;\langle x_{1},\;\ldots ,\;x_{u}\rangle \right\rangle$: *p*($w,\;t,\;\langle x_{1},\;\ldots ,\;x_{u}\rangle$)} in the conversational context $\left\langle w_{c},\;t_{c},\;\langle y_{1},\;\ldots ,\;y_{u}\rangle \right\rangle$ iff
  - *A* = $y_{n}\in \{y_{1},\;\ldots ,\;y_{u}\}$
  - there are relations *R*${}_{1}$ … *R*${}_{u}$ such that in $w_{c}$ at $t_{c}$, $y_{n}$ is uniquely *R*${}_{1}$-related to $y_{1}$, … , and $y_{n}$ is uniquely *R*${}_{u}$-related to $y_{u}$ (where *R*${}_{n}$ is the identity relation) and $y_{n}$'s standing in *R*${}_{1}$ … *R*${}_{u}$ to $y_{1}\ldots y_{u}$ establishes a conversation between $y_{1}\ldots y_{u}$
  - every multicentered world $\left\langle w^{\prime },\;t^{\prime },\;\langle x_{1}^{\prime },\;\ldots ,\;x_{u}^{\prime }\rangle \right\rangle$ compatible with what $y_{n}$ believes in $w_{c}$ at $t_{c}$ is such that *p*($w^{\prime },\;t^{\prime },\;\langle x_{1}^{\prime },\;\ldots ,\;x_{u}^{\prime }\rangle$) = 1. A multicentered world $\left\langle w^{\prime },\;t^{\prime },\;\langle x_{1}^{\prime },\;\ldots ,\;x_{u}^{\prime }\rangle \right\rangle$ is compatible with what $y_{n}$ believes in $\langle w_{c},\;t_{c},\;$ $\left\langle y_{1},\;\ldots ,\;y_{u}\rangle \right\rangle$ if $y_{n}$ believes in $w_{c}$ at $t_{c}$ that she might be the member $x_{n}^{\prime }$ of a group $\left\langle x_{1}^{\prime },\;\ldots ,\;x_{u}^{\prime }\right\rangle$ in $w^{\prime }$ at $t^{\prime }$ whose members are uniquely related by *R*${}_{1}$ … *R*${}_{u}$.

n-Belief in a conversational context entails that Lingens 1-believes *TIRED_1_* in the conversational context $\langle w_{c},\;t_{c},\;\langle$Lingens, Ortcutt⟩⟩ just in case (i) Lingens ∈ {Lingens, Ortcutt}, (ii) there are conversation-establishing relations *R* ${}_{1}$, *R* ${}_{2}$ and *R* ${}_{2}$ such that in $w_{c}$ at $t_{c}$ *R* ${}_{1}$ (identity) uniquely relates Lingens to himself and *R* ${}_{2}$ (the addressing relation) relates Lingens to Ortcutt, and (iii) every multicentered world $\left\langle w^{\prime },\;t^{\prime },\;\langle x_{1}^{\prime },\;x_{2}^{\prime }\rangle \right\rangle$ compatible with what Lingens believes in $w_{c}$ at $t_{c}$ is such that $x_{1}^{\prime }$ is tired of reading in $w^{\prime }$ at $t^{\prime }$.^24^

Let me briefly comment on the definition's formalism for conversational contexts, $\left\langle w_{c},\;t_{c},\;\langle y_{1},\;\ldots ,\;y_{u}\rangle \right\rangle$. If an agent *a* is in a conversation with just one person *b*, the conversational context will contain all and only *a* and *b*. The conversational context $\left\langle w_{c},\;t_{c},\;\langle a,\;b\rangle \right\rangle$ then *fixes* the conversational-establishing relations *R*, since the possible world $w_{c}$ at the time $t_{c}$ determines this information about the conversation, just as it determines every fact about the world at the time of the conversation. Importantly, the formalism does not tie the conversational roles of speaker and hearer to positions in the sequence. It may be that $\left\langle w_{c},\;t_{c},\;\langle a,\;b\rangle \right\rangle$ models a context in which *a* is speaking and $\left\langle w_{c},\;t_{c+1},\;\langle a,\;b\rangle \right\rangle$ models the conversation's context 1 moment later where *b* is speaking. We will come back to this in Section 8.

Abandoning technical details altogether for a moment, here is another example for illustration. You say to me, ‘I'm going to get coffee for us’, and I believe you. Then we both have a belief with the same content – very roughly, the set of pairs with one member about to get coffee for both members. But for you to believe that content is for you to believe it in a way that makes you the coffee-buying member. For me to believe it is to believe it in a way that makes me the coffee-receiving member. That's why (among other things), you stand up and make your way to the counter, and I stay put and wait for my coffee.

There is nothing mysterious about an agent's believing a multicentered worlds content in a conversational context, once we accept centered worlds content and individual self-location. We can, if we want, translate multicentered worlds belief into centered worlds belief. Put simply, to ascribe a property to the group of which one is a member is equivalent to self-ascribing the property of being a member of a group that has this property. For Ortcutt to believe the multicentered worlds content *p* expressed by Lingens' assertion of ‘I am tired of reading’ in the conversational context $\langle w_{c},\;t_{c},\;\langle$Lingens, Ortcutt⟩⟩ is for him to be addressed by Lingens and to believe the centered worlds content *p'* = {⟨w,t,x⟩: there is a *y*, *x* is *y*'s addressee in *w* at *t*, and *p*(w,t,⟨y,x⟩)}. More generally, the following equivalence holds:

Multicentered worlds belief and centered worlds belief

An agent *A* *n*-believes a multicentered worlds content {$\left\langle w,\;t,\;\langle x_{1},\;\ldots ,\;x_{u}\rangle \right\rangle$: *p*($w,\;t,\;\langle x_{1},\;\ldots ,\;x_{u}\rangle$)} in the conversational context $\left\langle w_{c},\;t_{c},\;\langle y_{1},\;\ldots ,\;y_{u}\rangle \right\rangle$ iff

- *A* = $y_{n}\in \{y_{1},\;\ldots ,\;y_{u}\}$,
- there are relations *R*${}_{1}$ … *R*${}_{u}$ such that in $w_{c}$ at $t_{c}$, $y_{n}$ is uniquely *R*${}_{1}$-related to $y_{1}$, … , and $y_{n}$ is uniquely *R*${}_{u}$-related to $y_{u}$ (where *R*${}_{n}$ is the identity relation) and $y_{n}$'s standing in *R*${}_{1}$ … *R*${}_{u}$ to $y_{1}\ldots y_{u}$ establishes a conversation between $y_{1}\ldots y_{u}$
- *A* believes the centered worlds content {⟨w,t,x⟩: there are individuals $x_{1},\;$ …, $x_{u}$ such that *x* is uniquely *R*${}_{1}$-related to $x_{1}$, *x* is uniquely *R*${}_{2}$-related to $x_{2}$, …, and *x* is uniquely *R*${}_{u}$-related to $x_{u}$ in *w* at *t*, and *p*($w,\;t,\;\langle x_{1},\;\ldots ,\;x_{u}\rangle$)}.

*N*-believing and *m*-believing in a conversational context are two different doxastic states (for *n* ≠ *m*), with different potential effects on action. If Lingens and Ortcutt communicate successfully, both come to have beliefs with the same multicentered worlds content *p*. However, in the conversational context $\langle w_{c},\;t_{c},\;\langle$Lingens, Ortcutt⟩⟩, Lingens will come to 1-believe *p*, which probably disposes him to stop reading, while Ortcutt will come to 2-believe *p*, which will not dispose him to such action. Multicentered worlds belief and centered worlds belief makes this evident: When Lingens 1-believes and Ortcutt 2-believes *p*, they believe the same content *p*, but for each the centered worlds belief equivalent to his belief in *p* is different.

A word of clarification on Multicentered worlds belief and centered worlds belief. The equivalence between multicentered worlds belief and centered worlds belief can be read in three ways. First, as stating an equivalence between two equally basic and theoretically useful notions of belief. Second, as stating a reductive explanation of believing a multicentered worlds content in terms of believing a centered worlds content. The fundamental notion of belief then is individual self-location. Call this option conservative multicentering.^25^ Third, the equivalence can be read as stating a reductive explanation of belief in centered worlds content in terms of belief in multicentered worlds content. The fundamental notion of belief then is collective self-location, or group-location. On this third option, individual self-location is a limiting case of collective self-location where the group consists of only one member. Call this view radical multicentering. I am inclined to think that radical multicentering is right. It has two closely related virtues: First, taking multicentered worlds belief and content as fundamental supports the Lockean assumption that communication essentially involves the sharing of a single content.^26^ If centered worlds belief is fundamental and speaker and hearer in successful communication have beliefs with different centered worlds contents, introducing a shared content at the non-fundamental level of multicentered worlds belief hardly goes a long way towards saving the Lockean picture. Second, radical multicentering makes sense of the notion of a conversation's common ground – the set of contents presupposed by all interlocutors (cf. Stalnaker 1978). The common ground contains multicentered worlds contents, which are presupposed by all interlocutors. On conservative multicentering, in contrast, the common ground is reduced to a number of sets of centered worlds contents, one set of presuppositions for each interlocutor. We could call this a common ground, but the commonalities between interlocutors' presuppositions are a lot more indirect than on radical multicentering's conception of the common ground as a single set of contents. I will return to the discussion of the common ground in Section 9.^27^

## 7. A semantics for pronouns and predicates of personal taste

Understanding Speech act content in terms of multicentered worlds was the key to solving the *de se* and incompatibility problems in a way that reconciles the self-locating account of belief with the Lockean picture of communication. But so far I have merely claimed that Speech act content is multicentered worlds content. I have not yet shown what the relation is between sentences – such as ‘This cookie is tasty’ and ‘I am hungry’ – and this kind of speech act content. For the multicentered worlds model to be plausible, it needs to be completed with a semantics of predicates of personal taste and of personal pronouns and an account of how the semantics determines multicentered worlds speech act content.

The multicentered worlds model does not require a radical departure from existing semantic proposals. For instance, the standard Kaplanian treatment of personal pronouns can be combined with a slightly modified version of Stephenson's (2007a) semantics for predicates of personal taste to make room for multicentered worlds content. Other options are available, but for concreteness I will introduce a combination of the above in this section.^28^ In the next section, I will show how this semantics delivers multicentered worlds speech act content.

Our starting point is a Kaplan-style intensional semantic theory on which extensions are assigned to expressions relative to a context *c* and an index *i*.^29^ An expression's semantic value is a function from a context and an index to an extension; we will also say that an expression's semantic value *at a context and index* is an extension. A context *c* is a possible occasion of use of an expression, which determines at least a world, a time, a speaker, addressee(s), and a location. Formally, we will represent a context as a multicentered world $\left\langle w,\;t,\;\langle x_{1},\;\ldots ,\;x_{n}\rangle \right\rangle$ such that $x_{1},\;\ldots ,\;x_{n}$ are in a conversation with each other in *w* at *t*. An index *i* is a sequence of independently shiftable features of context, called coordinates. In the semantics we need, the index is a triple $\left\langle w,\;t,\;\langle x_{1},\;\ldots ,\;x_{n}\rangle \right\rangle$.^30^ The index is the first modification of Stephenson's system, which has ⟨w,t,x⟩-triples as indices. The double brackets ‘⟦  ⟧’ denote the interpretation function, a three-place function that maps an expression, a context and an index to an extension.

The extensions of standard one-place predicates like ‘hungry’ depend on the world- and time-coordinate of the index, but not on any individual in the sequence.

(10) ⟦hungry⟧${}^{c,\;i}$ = [*λ*y${}_{e}$. y is hungry in *w* at *t*],
  for *i* = $\left\langle w,\;t,\;\langle x_{1},\;\ldots ,\;x_{n}\rangle \right\rangle$^31^

Predicates of personal taste (PPTs) such as ‘tasty’ and ‘fun’ are two-place predicates on Stephenson's view.^32^ They are functions from an experiencer and experienced object or individual, and a context and index, to a truth value.

(11) ⟦tasty⟧${}^{c,\;i}$ = [*λ*y${}_{e}$.[*λ*z${}_{e}$. z tastes good to y in *w* at *t*] ]
  ⟦fun⟧${}^{c,\;i}$ = [*λ*y${}_{e}$.[*λ*z${}_{e}$. z is fun for y in *w* at *t*] ]

The entries for PPTs do not directly make their extensions dependent on the individuals in the index. However, in first-personal uses of PPTs – i.e. in bare taste claims such as ‘This cookie is tasty’ which are based on the asserter's taste preferences but which do not have an overt experiencer argument in the sentence's surface structure – there is a covert, phonologically null nominal item ‘PRO_C_’ at the appropriate level of Logical Form.^33^ Simplifying the Logical Form, and ignoring tense and the contribution of the copula, (12) has the following structure.

(12) This cookie is tasty.
  [This cookie ] [ is tasty PRO_C_ ]

PRO_C_ takes as its referent the sequence of centers in the index:

(13) ⟦PRO_C_⟧${}^{c,\;i}$ = $\left\langle x_{1},\;\ldots ,\;x_{n}\right\rangle$,
  where the index *i* = $\left\langle w,\;t,\;\langle x_{1},\;\ldots ,\;x_{n}\rangle \right\rangle$.

PRO_C_ is the second modification of Stephenson's system, in which the nominal item PRO_J_ refers to the single judge given by the index. Like PRO_J_, PRO_C_ is ‘not a pronoun in the sense of being able to be bound or controlled, nor is it an indexical since it takes its reference from the index rather than the context of utterance’ (Stephenson 2007a, 500). PRO_C_ thus introduces dependence of first-personal uses of PPTs on the individuals in the index into the system.^34^ The meaning of (12) is computed in (14).^35^

(14) ⟦This cookie is tasty⟧${}^{c,\;i}$ =
  ⟦tasty⟧${}^{c,\;i}$ (⟦PRO_C_⟧${}^{c,\;i}$) (⟦this cookie⟧${}^{c,\;i}$) = 1 iff
  ⟦tasty⟧${}^{c,\;i}$ ($\left\langle x_{1},\;\ldots ,\;x_{n}\right\rangle$) (the cookie) = 1 iff
  the cookie tastes good to $\left\langle x_{1},\;\ldots ,\;x_{n}\right\rangle$ in *w* at *t*

Personal pronouns like ‘I’/‘me’ and (2nd person singular) ‘you’ receive a standard Kaplanian treatment.

(15) ⟦I⟧${}^{c,\;i}$ = the speaker/agent of *c*
  ⟦you⟧${}^{c,\;i}$ = the addressee of *c*

We represent contexts by multicentered worlds, so the entries for these pronouns are to be understood as follows. In $c=\langle w_{c},\;t_{c},\;\langle x_{1},\;\ldots ,\;x_{n}\rangle$, ‘I’ refers to the $x_{i}$ of $\left\langle x_{1},\;\ldots ,\;x_{n}\right\rangle$ that plays the speaker role in $w_{c}$ at $t_{c}$; ‘you’ refers to the $x_{i}$ of $\left\langle x_{1},\;\ldots ,\;x_{n}\right\rangle$ that plays the role of addressee in *c*. Ben's utterance in (16) has the meaning given in (17).

(16) Ben: I am hungry.
(17) ⟦I am hungry⟧${}^{c,\;i}$ = ⟦hungry⟧${}^{c,\;i}$ (⟦I⟧${}^{c,\;i}$) = 1 iff
  ⟦hungry⟧${}^{c,\;i}$ (Ben) = 1 iff
  Ben is hungry in *w* at *t*

We now have what we need for a compositional semantic theory for simple sentences expressing claims about taste and *de se* attitudes.^36^

## 8. Speech act content

How do we get the multicentered worlds speech act content we need from the semantic values given above? The short answer is: by taking the diagonal of a sentence's Kaplanian character. Let us start with sentences expressing *de se* attitudes.

Kaplan took ‘what is said’ – the speech act content expressed – by an assertoric utterance of a sentence in context to be the function from index to truth values. Let us call this content, in our system, the Kaplan horizontal:

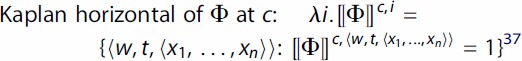

Given the standard Kaplanian semantic values of the pronouns ‘I’/‘me’ and ‘you’, their reference is resolved in the derivation of the Kaplan horizontal from context. Thus, the Kaplan horizontal of (16) is (18).

(18) λi⟦I am hungry⟧${}^{c,\;i}$ = {$\left\langle w,\;t,\;\langle x_{1},\;\ldots ,\;x_{n}\rangle \right\rangle$: Ben is hungry in *w* at *t*}

But (18) is not the interesting multicentered worlds content which, as we saw above, is communicated by assertions involving 1st-personal pronouns. Fortunately, the Kaplan horizontal is not the only content definable from the semantics. As Lewis (1980) showed, the intensional semantic theory does not determine one unique candidate for the role of speech act content. To be sure, it is convenient if the content that is the input to intensional operators – i.e. here the Kaplan horizontal – is also the content expressed in communication. But speech act content *need not* be identical to the content that combines with intensional operators to yield a sentence's semantic value in context.^38^ It is this freedom that allows us to define the right interesting multicentered worlds content from the semantic value of sentences containing 1st-personal pronouns.

Suppose again that Ben utters ‘I am hungry’ in the conversational context $\langle w^{\prime },\;t^{\prime },\;\langle$Ben, Anna⟩⟩. As we saw above, the interesting multicentered worlds content he communicates is *HUNGRY_1_*, repeated in (19).

(19) *HUNGRY_1_*: {$\left\langle w,\;t,\;\langle x_{1},\;x_{2}\rangle \right\rangle$: $x_{1}$ is hungry in *w* at *t*}.

*HUNGRY_1_* is, near enough, the Kaplan diagonal of the sentence ‘I am hungry’ as asserted by Ben. The Kaplan diagonal of a sentence Φ is the set of contexts at which Φ is true.


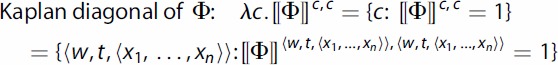


Recall that a context *c* is modeled by a multicentered world. So the Kaplan diagonal is a multicentered worlds content. Recall also that for every conversational situation, there are several equivalent multicentered worlds representations of that situation, which only differ in the order of the individuals in the sequence. Since for Ben's speech situation, we have represented the context by $\langle w^{\prime },\;t^{\prime },\;\langle$Ben, Anna⟩⟩ – in which Ben, the speaker, comes first – the resulting choice for the Kaplan diagonal is one in which the speaker-center is the first in the sequence. Ben's utterance of ‘I am hungry’ is true at all contexts in which the speaker is hungry, which given the choice of context-representation is just the set of contexts $\left\langle w,\;t,\;\langle x_{1},\;x_{2}\rangle \right\rangle$ such that $x_{1}$ is hungry in *w* at *t*. So the Kaplan diagonal of Ben's assertion in a conversation with one addressee is *HUNGRY_1_*, as required.

But we need to do a little more work to get *HUNGRY_1_* as Ben's speech act content. The challenge is the following: for any situation, or context, there are many formal contexts that represent it and which differ only in the order of the individuals in their sequence. For the situation in (16) in which Ben utters ‘I am hungry’, we have the two multicentered worlds $\langle w^{\prime },\;t^{\prime },\;\langle$Ben, Anna⟩⟩ and $\langle w^{\prime },\;t^{\prime },\;\langle$Anna, Ben⟩⟩. Yet we do not want, for any situation in which the speaker is hungry, both multicentered worlds representing the situation to be members of *HUNGRY_1_*, or else we would not individuate the *first (and only the first*) center-slot's hungriness. If both multicentered worlds representing a given situation went into the speech act content, we would always get contents indiscriminately attributing properties to every center (if to any). The challenge is to integrate the account of speech act content with the semantics of pronouns in a way that allows only the ‘right’ multicentered world for a situation to be a member of the Kaplan diagonal.

The challenge can be met if we choose one multicentered world for the representation of the context of utterance – the conversational context – and allow only those multicentered worlds into the speech act content which assign the conversational roles of speaker, addressee etc. to the individuals in the same respective positions in the sequence as the conversational context does. When applied to Ben's assertion of ‘I am hungry’ in (16), this means: The Kaplanian character of ‘I’, given in (15) above, is the rule to pick out the context's speaker. If we choose $\langle w^{\prime },\;t^{\prime },\;\langle$Ben, Anna⟩⟩ for Ben's assertion, ‘I’ picks out the first center of the sequence. So for any two-persons situation in which the speaker is hungry, we need that multicentered world to be in *HUNGRY_1_* in which the first center is the speaker.

To generalize this solution, it will be helpful to have the notion of a ‘canonical context relative to a context of utterance’: In any set of contexts that differ only in the order of the individuals in their sequence, the canonical context relative to the context of utterance is that context in which the center-slots occupy the same conversational roles as the center-slots of the context of utterance. Let us make this more precise by introducing a few definitions.

A *conversation *C** is represented by a triple $\left\langle w,\;\langle t_{1},\;\ldots ,\;t_{m}\rangle ,\;\{x_{1},\;\ldots ,\;x_{n}\}\right\rangle$ of a world *w* in which the conversation takes place, a sequence of moments $t_{1}$ … $t_{m}$ at which the conversation takes place, and an unordered set of individuals $x_{1},\;\ldots ,\;x_{n}$ which are the conversation's participants.

For every conversation *C* = $\left\langle w,\;\langle t_{1},\;\ldots ,\;t_{m}\rangle ,\;\{x_{1},\;\ldots ,\;x_{n}\}\right\rangle$, we can pick a sequence $\left\langle x_{1},\;\ldots ,\;x_{n}\right\rangle$ that is the *canonical sequence of *C**.

Then we can say that a context $\left\langle w_{c},\;t_{c},\;\langle x_{1}^{c},\;\ldots ,\;x_{n}^{c}\rangle \right\rangle$ is a *context of the conversation *C**, $\left\langle w_{1},\;\langle t_{1},\;\ldots ,\;t_{m}\rangle ,\;\{x_{1},\;\ldots ,\;x_{n}\}\right\rangle$, just in case (i) $w_{c}=w_{1}$, $t_{c}\in \{t_{1},\;\ldots ,\;t_{m}$}, and $x_{1}^{c},\;\ldots ,\;x_{n}^{c}\in \{x_{1},\;\ldots ,\;x_{n}\}$. A context of the conversation *C* is any multicentered world that represents a moment in the conversation *C*.

A context $\left\langle w_{c},\;t_{c},\;\langle x_{1}^{c},\;\ldots ,\;x_{n}^{c}\rangle \right\rangle$ is a *canonical context of the conversation *C** just in case (i) it is a context of *C* and (ii) the context's sequence $\left\langle x_{1}^{c},\;\ldots ,\;x_{n}^{c}\right\rangle$ is the canonical sequence of *C*.

In every context $\left\langle w_{c},\;t_{c},\;\langle x_{1}^{c},\;\ldots ,\;x_{n}^{c}\rangle \right\rangle$, each member of the sequence $x_{1}^{c},\;\ldots ,\;x_{n}^{c}$ occupies a *conversational role*. At least the following are conversational roles: speaker, hearer, and (intended 2nd-person) addressee(s).

We can now define the notion of a *canonical context relative to $c_{U}$*: A context *c* = $\left\langle w_{c},\;t_{c},\;\langle x_{1}^{c},\;\ldots ,\;x_{n}^{c}\rangle \right\rangle$ is a canonical context relative to $c_{U}$, $\left\langle w_{c_{U}},\;t_{c_{U}},\;\langle x_{1}^{c_{U}},\;\ldots ,\;x_{n}^{c_{U}}\rangle \right\rangle$, just in case (i) $c_{U}$ is a canonical context of a conversation *C* and (ii) every $x_{i}^{c}$ plays the same conversational role in *c* that $x_{i}^{c_{U}}$ plays in $c_{U}$.

Note that contexts as defined here differ from Kaplan's formal definition of context, since Kaplan ties conversational roles to the positions of individual coordinates in his ordered tuples but we don't. For different speech acts in the same conversation *C*, we model them with canonical contexts of *C* and thereby ensure that in each of those contexts, the order of the sequence of individuals remains the same. In conversational turn-taking, speaker and hearer roles switch between interlocutors. So from one canonical context to another of the same conversation, it may differ which individual in the sequence occupies which conversational role. In this way, conversational roles are not tied to positions in the sequence. Still, our contexts fix conversational roles, since this information is determined by the possible world $w_{c}$ and time $t_{c}$ of the context.

We can now rule out the ‘wrong’ contexts in our account of speech act content – those in which conversational roles are assigned to center-slots in a different order than in the context of utterance – by stipulating that the Kaplan diagonal of any speech act contain only canonical contexts relative to the context of utterance. The definition of speech act content is as follows:

Speech act content_MW_

The content of an assertion of Φ in *c* is the Kaplan diagonal of Φ in *c*, which consists only of canonical contexts relative to *c*.

Consider once more Ben's assertion of ‘I am hungry.’ Let us say that the canonical context of his assertion in his conversation is $\langle w^{\prime },\;t^{\prime },\;\langle$Ben, Anna⟩⟩, as before. So in the assertion's canonical context, Ben in the first sequence-slot occupies the conversational role of speaker. For any pair of contexts $\left\langle w,\;t,\;\langle x_{1},\;x_{2}\rangle \right\rangle$ and $\left\langle w,\;t,\;\langle x_{2},\;x_{1}\rangle \right\rangle$ in which the speaker is hungry, only the one in which the individual in the first sequence-slot occupies the role of speaker is a canonical context relative to $\langle w^{\prime },\;t^{\prime },\;\langle$Ben, Anna⟩⟩ and may be in the Kaplan diagonal of the assertion. As a result, the speech act content is *HUNGRY_1_*: {$\left\langle w,\;t,\;\langle x_{1},\;x_{2}\rangle \right\rangle$: $x_{1}$ is hungry in *w* at *t*}.

Speech act content_MW_ also yields the desired content for first-personal taste claims. Such taste claims put conditions on every center of the sequence. Since the experiencer argument PRO_C_ takes its value from the index, the intension (function from index to extension) of a sentence involving PPTs on first-personal uses does not vary from context to context.^39^ The speech act content of ‘Liquorice is tasty’ is (20).^40^

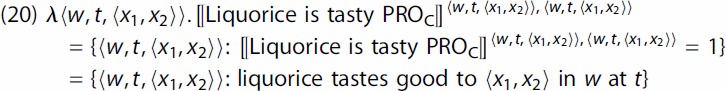

## 9. Conversation and the common ground

On the multicentered worlds model, Speaker's mental content, Speech act content, and Hearer's mental content are one and the same multicentered worlds content. Thus Belief-speech coordination and Speech-belief coordination are given by the identity of these contents. The basic Lockean idea that one piece of information travels from speaker's head to hearer's head is preserved.

The multicentered worlds model fits naturally with a Stalnakerian implementation of the Lockean picture. I will first sketch Stalnaker's original account and then make the changes needed to accommodate multicentered worlds content.

According to Stalnaker, linguistic communication is primarily a matter of updating and establishing a body of shared information – the common ground.^41^ Speech acts serve to influence this body of information in various ways. In particular, the essential effect of assertion is to add the asserted content to the common ground. The attitude that speakers strike towards the common ground is the attitude of *presupposition*:

 … the presuppositions of a speaker are the propositions he takes for granted as part of the background of the conversation. A proposition is presupposed if the speaker is disposed to act as if he assumes or believes that the proposition is true, and as if he assumes or believes that his audience assumes or believes that it is true as well. (Stalnaker 1978, 84)

Presupposition, in this sense, is a public attitude: one presupposes a proposition *p* only if one presupposes that everyone else in the conversation also presupposes *p*. A speaker's presuppositions are represented by the speaker's *context set*: the set of possible worlds compatible with what the speaker presupposes (Propositions, for Stalnaker, are also sets of possible worlds; a speaker's context set is the intersection of the propositions she presupposes.) There is a context set for each participant in a conversation, but when things go as they should, all participants make the same presuppositions and the speakers' context sets coincide with the *conversation's context set*. The common ground is represented by the conversation's context set, which is the intersection of the propositions in the common ground. A conversation is *defective* when the conversation's participants do not all make the same presuppositions.^42^

Assertions are proposals to add information to the common ground. When an assertion of *p* is understood and accepted by all participants in a conversation, its content *p* becomes presupposed in the conversation, and its effect is to eliminate all the non-*p* worlds from the conversation's context set. ‘To engage in conversation is, essentially, to distinguish among alternative possible ways that things may be ’ (Stalnaker 1978, 85). An assertion's primary contribution is to narrow down what the participants commonly take to be the possible relevant ways the world might be.

This is, in bare outline, Stalnaker's picture of assertion and communication. On the multicentered worlds model, the conversation's context set is a set of multicentered worlds whose sequences have as many centers as the conversation has participants. To engage in conversation is to distinguish between alternative ways that the conversational participants might be, where this does not require that they all share the ways they *individually* might be. Intuitively, the purpose of conversation is the coordination of individual perspectives, sometimes with the result of sharing a perspective, sometimes with the result of having one's individual perspective noticed.^43^

Assertions serve this purpose, if successful, by adding the multicentered worlds content they express to the common ground. When in the common ground, that content is presupposed by all conversational participants. We can define the notion of speaker presupposition for a context set containing multicentered worlds on the basis of *n*-belief in a conversational context:

Speaker presupposition_MW_

A speaker *S* *n*-presupposes a multicentered worlds content *p* in a conversational context $\left\langle w_{c},\;t_{c},\;\langle x_{1},\;\ldots ,\;x_{u}\rangle \right\rangle$ iff *S* = $x_{n}\in \{x_{1},\;\ldots ,\;x_{u}\}$ and *S* is disposed to act as if she *n*-assumes or *n*-believes *p*, and as if she *n*-assumes or *n*-believes that for all $x_{i}\in \{x_{1},\;\ldots ,\;x_{u}\}$, $x_{i}$ *i*-assumes or *i*-believes *p* as well.

If a multicentered worlds content *p* is part of the common ground in the default case where the common ground is common belief, every participant $x_{n}$ in the conversation *n*-believes *p*.

The Stalnakerian model with multicentered worlds content vindicates the Lockean idea that one content is what is expressed by the speaker and believed by all participants in successful communication. At the same time, belief on the model still is a form of self-location, although belief in a conversational context involves locating not just oneself but the conversational group of which one is a member. The cognitive differences between different participants' beliefs in the same multicentered worlds content surface in the way they self-and-group-locate, which we capture by relativizing belief to the believer's position in the conversational sequence. The model solves the *de se* problem and the incompatibility problem by keeping centers and individual possibilities separate where necessary and by allowing for joint possibilities to be established where this is, intuitively, the effect of successful assertion. Thus, the conflict between the Lockean picture of communication and the self-locating belief model can be resolved without giving up either of them.

## 10. Norms of assertion

We have said enough about communication on the multicentered worlds model to establish the simple links between Speaker's mental content, Speech act content, and Hearer's mental content on the Lockean picture of communication. But we have not established that the multicentered worlds model makes the right predictions about the assertability and acceptability of *de se* assertions and assertions about matters of taste. Our model of communication should tell us under which conditions it is felicitous for speakers to assert a multicentered worlds content *p*, and when it is a good idea for hearers to accept *p* into the common ground. In what follows, I will introduce norms of assertion and acceptance by focusing on the pragmatics of discourse about subjective matters. In the next section, I will discuss an objection to the multicentered worlds view of the communication of subjective attitudes.

So what are the norms of assertion and acceptance on the multicentered world view? Clearly, egocentric norms do not make the right predictions. To see this, let us consider one mainstream approach to norms of assertion, which states the crucial necessary condition for felicitous assertion in terms of truth of the asserted content. First, it will be helpful to distinguish between two kinds of perspectives at which a content may be true.

Individual perspective P^1^ = ⟨w,t,x⟩

An individual perspective *P^1^* represents the perspective of a single individual (her and only her location and world view) in the world *w* at the time *t*.

The conversation's perspective P^n^ = $\left\langle w_{c},\;t_{c},\;\langle x_{1},\;\ldots ,\;x_{n}\rangle \right\rangle$

The perspective of a conversation at $t_{c}$ is $\left\langle w_{c},\;t_{c},\;\langle x_{1},\;\ldots ,\;x_{n}\rangle \right\rangle$, where $w_{c}$ is the world at which the conversation takes place, $t_{c}$ is a moment in the conversation, and $\left\langle x_{1},\;\ldots ,\;x_{n}\right\rangle$ is the conversational sequence for the conversation. $\left\langle w_{c},\;t_{c},\;\langle x_{1},\;\ldots ,\;x_{n}\rangle \right\rangle$ represents the individual perspectives of all conversational participants $x_{1},\;\ldots ,\;x_{n}$ in $w_{c}$ at $t_{c}$.

Let us say that a multicentered worlds content is true from an individual perspective just in case it correctly represents the location of that individual – no matter the location of the other individuals in the sequence. We can then give the following egocentric truth norm of assertion:

Egocentric Truth Norm

**Table:** 

Assert_E_	A speech act content *p* is appropriately assertable in context *c* only if *p* is true from the speaker's individual perspective *P_c_^1^* in *c*.

Assert_E_ may seem to make the right predictions for *de se* assertions. Intuitively, Heimson may assert ‘I am Heimson’ only if the center-slot representing Heimson correctly locates him in the world at the time. But Assert_E_ fails to make the correct predictions for *de te* assertions. It does not prohibit speakers to tell anyone except Heimson, ‘You are Heimson', as it should. The speech act content expressed by ‘You are Heimson’ places a constraint only on a center different from the center representing the speaker. As long as someone in the world and at the time of the conversation is Heimson, the speech act content is true from the speaker's individual perspective.

The right norms of assertion and acceptance, which go hand in hand with belief in context as group-location, are group-centric norms. In a first attempt, we can formulate them as follows.

Group-centric truth norms (1st attempt)

**Table:** 

Assert_G_	A speech act content *p* is assertable in context *c* only if *p* is
	true from the conversation's perspective in *c*.
	
Accept_G_	A speech act content *p* is acceptable in context *c* if *p* is true
	from the conversation's perspective in *c*.

Suppose Ben tells Heimson in a context of utterance *c*, ‘You are mad.’ Let the conversational context be $\langle w_{c},\;t_{c},\;\langle$Ben, Heimson⟩⟩ so that the speech act content of Ben's assertion is {$\left\langle w,\;t,\;\langle x_{1},\;x_{2}\rangle \right\rangle$: $x_{2}$ is mad in *w* at *t*}. The conversation's perspective *P_c_^ 2^* is $\langle w_{c},\;t_{c},\;\langle$Ben, Heimson⟩⟩. According to Assert_G_, Ben's assertion is appropriate only if {$\left\langle w,\;t,\;\langle x_{1},\;x_{2}\rangle \right\rangle$: $x_{2}$ is mad in *w* at *t*} is true from $\langle w_{c},\;t_{c},\;\langle$Ben, Heimson⟩⟩. This is as it should be. Ben should make the assertion only if – and Heimson should accept the assertion if – Heimson is mad at the time and world of speaking.^44^

For talk about taste, the group-centric norms entail that, for instance, ‘This cookie is tasty’ is assertable only if the cookie tastes good to speaker *and audience*. That is because all interlocutors have to be correctly located in the content, which says of each one of them that the cookie is tasty to them.

This prediction might seem too strong. Why should a speaker have to make sure that she and her audience have a common outlook on taste in order to guarantee that her assertion about the cookie's tastiness is appropriate? It may seem after all that the subjectivity of taste claims is better captured by an egocentric norm like Assert_E_. Yet we have also seen that egocentric norms deliver wrong results for *de te* assertions.

I will argue that the intuitive judgments motivating a weaker requirement on the appropriateness of bare taste assertions can be given their due place on the multicentered worlds view without denying that the above group-centric norm plays an important role in governing bare taste assertions in conversation.^45^ As I will show momentarily, two norms of assertion – a strong group-centric norm and a weak speaker-oriented norm – hold sway over discourse, each related to a different conversational purpose. The basic picture is this. Conversations are cooperative enterprises with the goal of locating the conversational group, i.e. reducing the group-possibilities in the context set. When bare taste assertions are made, this goal requires that participants agree on the tastiness of the food in question (or agree to disagree). But while the maximally cooperative, group-centric communicative purpose of bare taste claims is to establish a shared perspective on the tastiness of the food, bare taste claims also serve the more speaker-oriented purpose of giving voice to the speaker's own perspective. Each of these two purposes gives rise to a norm of assertion, which is conditional on the purpose. Judgments about the appropriateness of assertions may reflect either of the norms, depending on the purpose guiding the judgment.

The plan for the rest of this section is as follows. I will first show what explanatory work is done by the strong group-centric norm of assertion. I will then turn to intuitive judgments about the appropriateness of bare taste assertions that are weaker than those guided by the strong norm. This will require discussing the expressive-persuasive nature of bare taste assertions and how it is accounted for on the multicentered worlds model. At the end of the section, we will be in a position to state the two norms of assertion.

Let us start with the strong group-centric norm of assertion, Assert_G_, and the conversational goal of establishing a shared perspective on the tastiness of a food in question. Disputes about taste often become unreasonable when it is clear that no agreement can be reached. There is a sense in which bare taste claims, but not explicitly relativized taste claims, are pointless and uncooperative conversational moves when it has already been established in conversation that speaker and audience do not share tastes. It is often, but not always, unreasonable to keep insisting that some food is tasty when one's interlocutor has made it plain that she strongly disagrees with that judgment. In this kind of situation, however, it *is* reasonable to retreat to the claim that the food is tasty to oneself. For illustration, consider the following example.

**Table:** 

(21)	a.	Ben:	Schnitzel is tasty.
	b.	Anna:	No, it's not tasty! It is bland.
	c.	Ben:	Well, it's tasty to *me*, at least.

**Table:** 

(22)	a.	Ben:	Schnitzel is tasty.
	b.	Anna:	No, it's not tasty! It is bland.
	c.	Ben:	? Well, it is tasty.

The strong group-centric norm Assert_G_, but no egocentric norm, explains the difference in assertability between (21c) and (22c).^46^ Egocentric norms predict that both (21c) and (22c) are felicitous, since from Ben's perspective at the time of his second utterance, it is both true that Schnitzel is tasty and that Schnitzel is tasty to Ben himself.^47^ In contrast, the group-centric norm predicts that (22c) is not appropriate to assert in this kind of situation because the asserted content is not true from the conversation's perspective. But it makes no such prediction for (21c) because the asserted content – the set of pair-centered worlds such that Schnitzel tastes good to the speaker-center – is true from the conversation's perspective in the case where Schnitzel tastes good only to Ben. The group-centric norm captures the reasonableness of bare taste assertions, because their appropriateness conditions reflect the conditions of fully cooperative communicative success, which consists in an update of the common ground that entails that all interlocutors agree on the tastiness of Schnitzel. When in such situations we judge that a bare taste assertion is uncooperative and inappropriate, our judgments are guided by the fully cooperative, group-centric communicative purpose of bare taste claims.

It is a consequence of the strong group-centric norm of assertion that if there is significant divergence in our views on matters of taste, many of our taste assertions are likely to be inappropriate. But very often, especially at the beginning of a conversation about matters of taste, it seems perfectly appropriate to make a bare taste claim such as ‘This cookie is tasty’, even when someone among our audience as a matter of fact disagrees. How can we explain such judgments of conversational appropriateness?

Bare taste claims are aimed at establishing a shared perspective. But they also serve the purpose of voicing our own individual perspective. Under normal circumstances, I want my audience to share *my* perspective, and for that I need to put my perspective out there, in the hope that my audience will agree. In many cases, this hope is well-founded. Our perspectives are very often very similar. It is very often reasonable to assume that we are alike in our perspectives on the tastiness of the food in question, be it because it is reasonable to assume that as humans, we share a basic physiological make-up, or because we are similar in our dispositions to enjoy foods according to their taste, or because we belong to a community of values whose members arrive at similar evaluative judgments due to normative pressure towards the coordination of attitudes. Even when there is resistance from my audience that indicates they do not share my perspective, it might be reasonable – up to a point – to sustain the assumption of relevant similarity because there is good reason to think that they might *come to* share my perspective as a result of the conversation. Where the purpose of voicing one's perspective – with an eye to persuading the audience to adopt the perspective – is in the foreground, assertions seem appropriate only if they correctly voice the speaker's perspective and there is some chance that the audience can be persuaded to adopt the perspective, at least for the purpose of the conversation. Appropriateness in this sense is captured by the weak norm of assertion that is tied to the more speaker-oriented purpose of voicing one's perspective.

Before we can state the weak norm of assertion, we need to get clearer on the expressive-suggestive nature of bare taste assertions. This requires making precise the assumption of relevant similarity on the multicentered worlds framework. For conversational participants to assume that they are similar with respect to their perspectives on the tastiness of some food is for them to presuppose that they have a *joint perspective* on the multicentered worlds content *p*, which says that the food is tasty.

Joint perspective on p

$\left\langle w,\;t,\;\langle x_{1},\;\ldots ,\;x_{n}\rangle \right\rangle$ is a joint perspective on a multicentered worlds content *p* iff for all individuals $x_{i}$ and $x_{j}$ ∈{$x_{1},\;\ldots ,\;x_{n}$}: either both
  $\left\langle w,\;t,\;\langle x_{1},\;\ldots ,\;x_{i},\;x_{j},\;\ldots ,\;x_{n}\rangle \right\rangle$ ∈p and $\left\langle w,\;t,\;\langle x_{1},\;\ldots ,\;x_{j},\;x_{i},\;\ldots ,\;x_{n}\rangle \right\rangle$ ∈p, or both
  $\left\langle w,\;t,\;\langle x_{1},\;\ldots ,\;x_{i},\;x_{j},\;\ldots ,\;x_{n}\rangle \right\rangle$ ∈¬p and $\left\langle w,\;t,\;\langle x_{1},\;\ldots ,\;x_{j},\;x_{i},\;\ldots ,\;x_{n}\rangle \right\rangle$ ∈¬p.^48^

For a pair-centered content *p*, this means that the multicentered world $\langle w_{1},\;t_{1},\;\langle$Ben, Anna⟩⟩ is a joint perspective on *p* just in case either both $\langle w_{1},\;t_{1},\;\langle$Ben, Anna⟩⟩ ∈p and $\langle w_{1},\;t_{1},\;\langle$Anna, Ben⟩⟩ ∈p, or both $\langle w_{1},\;t_{1},\;\langle$Ben, Anna⟩⟩ ∈¬p and $\langle w_{1},\;t_{1},\;\langle$Anna, Ben⟩⟩ ∈¬p. Where *p* is a content expressed by a bare taste claim, this intuitively says that Anna and Ben have a joint perspective on the tastiness of some food in $w_{1}$ at $t_{1}$ just in case the food tastes good either to both of them or to neither of them in $w_{1}$ at $t_{1}$. A *presupposition* (in the sense defined in Section 9) *of joint perspective on p* is in place in a conversation with participants $x_{1},\;\ldots ,\;x_{n}$ just in case the context set contains only joint perspectives on *p*. For a conversation between Ben and Anna this means that a presupposition of joint perspective on a pair-centered content *p* is in place just in case for every multicentered world $\left\langle w,\;t,\;\langle x_{1},\;x_{2}\rangle \right\rangle$ in the context set, either both $\langle w,\;t,\;\langle x_{1},\;x_{2}\rangle \rangle \in p$ and $\langle w,\;t,\;\langle x_{2},\;x_{1}\rangle \rangle \in p$ or both $\langle w,\;t,\;\langle x_{1},\;x_{2}\rangle \rangle \in \neg p$ and $\langle w,\;t,\;\langle x_{2},\;x_{1}\rangle \rangle \in \neg p$.

Provided that speakers know their own taste and the context set contains the conversation's perspective (the ‘actual’ multicentered world), an assertion will not be inappropriate (in either weak or strong sense) in a conversation in which a correct presupposition of joint perspective is in place.^49^

Let us now move on to the expressive-suggestive nature of bare taste assertions. It is crucial to realize that the point of bare taste assertions is never just to state one's perspective. We observed a difference between asserting ‘This cookie is tasty’ and ‘This cookie is tasty to me.’ The latter is a statement of one's perspective, and it can function as a ‘partial retraction’ of one's bare taste claim. The former cannot function in this way (cf. (21), (22) above). So what is it about the bare taste assertion that distinguishes it from the mere statement of one's perspective?

Emotivists and others have long noted that evaluative claims have a persuasive, or recommending, force.^50^ They recommend a particular attitude towards the object, event, or action in question. On the multicentered worlds model, it is not hard to see how this could be so. If Ben asserts that liquorice is tasty, he proposes to add to the common ground the content that liquorice tastes good to all participants. For his assertion to be appropriate (in the weak sense), a presupposition of joint perspective has to be in place. If no such presupposition is yet in place and liquorice does not taste good to the addressee, she faces a choice. She can either reject the claim or she can *accommodate* the presupposition of joint perspective. She accommodates the presupposition if she comes to be act as if she assumes or believes that food of the relevant kind either tastes good to both the speaker and herself or to neither of them (and comes to act as if she assumes or believes that the speaker believes this and so on). The kind of accommodation is just what accommodation of any type of presupposition (on the Stalnakerian model) amounts to, viz. adding the missing presupposition to the common ground.^51^

In some cases, where common ground is common belief, the hearer's accommodation amounts to her coming to sincerely believe that liquorice tastes good to both of them only if she changes her individual perspective on the tastiness of liquorice, thus bringing it about that the taste claim is true. It is a peculiarity of the multicentered worlds framework that adding the presupposition of joint perspective may in such cases involve changing one's own perspective. Thus, the persuasive force of bare taste assertions amounts to the potential need for accommodation on the hearers' part, which they can bring about by changing their perspectives.

In other cases, speakers and hearers need not take belief or knowledge as the basic attitude of their presuppositions. Speaker presupposition_MW_ (Section 9) only requires interlocutors to be disposed to act as if they assume or believe what is in the common ground. So to accommodate ‘liquorice is tasty’ minimally requires the hearer only to accept the asserted multicentered content into the common ground by coming to act as if she asssumes or believes it. In this way, accommodation need not require hearers to sincerely change their perspective on the tastiness of some food, merely to accept-for-the-purposes-of-the-conversation that the same perspective on the food is shared by everyone in the conversation.^52^

We can thus explain why even when it is understood that the audience disagrees with the speaker about the tastiness of some food, it may still be sensible for the speaker to insist on her judgment as long as she has reason to think that her audience is in a position to accommodate. And even when she has little reason to think that her audience will in fact accommodate, the practical pressure of having to coordinate her perspective with her hearers' perspectives – for instance, when they have to take a collective decision on which food to order for their party – may provide sufficient reason to insist on a bare taste claim in light of opposition.^53^

Let me summarize. Judgments about the propriety of bare taste assertions may be guided by different conversational purposes. On the one hand, they may be guided by maximal cooperativeness – a property an assertion possesses if everyone in the conversation can appropriately accept it. These judgments are accounted for by the strong group-centric norm of assertion. They track reasonableness – what a dispute about matters of taste lacks when ‘it makes no sense’ to keep disputing. On the other hand, judgments may track a much lower threshold of appropriateness. In that case, they are guided by the speaker-oriented purpose of voicing one's perspective and persuading one's audience to share one's perspective. An assertion counts as appropriate in this weaker sense only if it correctly represents the speaker's perspective and there is a chance that the hearers may be persuaded – that is, the hearers are in a position to accommodate the asserted content.

In talk about subjective matters, the changes an assertion proposes to make to the common ground may be appropriate relative to the speaker-oriented purpose, yet fail to be appropriate with respect to maximal cooperativeness. Judgments may be guided by the strong group-centric norm of assertion or by the weak speaker-oriented norm of assertion.

Strong group-centric norm of assertion

**Table:** 

Assert_G_	A speech act content *p* is appropriately assertable in context *c* only if *p* is true from the conversation's perspective in *c*.

Weak speaker-oriented norm of assertion

**Table:** 

Assert_W_	A speech act content *p* is appropriately assertable in context *c* only if *p* correctly locates the speaker in *c*, and the hearers are in a position to accommodate *p*.

A speech act content_MW *p* correctly locates a speaker *S* just in case *p* contains a triple consisting of the speaker's actual world @, her current time *t*, and a sequence with *S* in the position that represents *S* relative to the conversational sequence: Given the conversational sequence ⟨*S*,…⟩, there is a triple ⟨@,t,⟨ *S*,…⟩⟩ such that ⟨@,t,⟨ *S*,…⟩⟩∈ *p*.

## 11. An objection from ‘tasty to us’

Let me finally address an objection from the use of PPTs.

Objection. On the multicentered worlds model, an assertion of ‘X is tasty’ may have the same content as an assertion of ‘X is tasty to us’ made in the same context. For instance, in a conversation between two people assertions of ‘Liquorice is tasty’ and ‘Liquorice is tasty to us’ both express (23).

(23) {$\left\langle w,\;t,\;\langle x_{1},\;x_{2}\rangle \right\rangle$: liquorice tastes good to $\left\langle x_{1},\;x_{2}\right\rangle$ in *w* at *t*}

But intuitively, an assertion of ‘Liquorice is tasty to us’ is about what tastes good to the group, whereas an assertion of ‘Liquorice is tasty’ is not. This difference shows in the different assertability conditions of the assertions. For instance, in a conversational context in which it is common belief that liquorice fails to taste good to at least one of the addressees, the assertion ‘Liquorice is tasty to us’ seems infelicitous. In contrast, a speaker to whom liquorice tastes good can still felicitously assert ‘Liquorice is tasty’ in that context. This strongly suggests that the two assertions have different truth conditions.

Reply. It will be helpful to first restate the objection in a rigorous way. I will then make a clarificatory remark before I explain why the two assertions may *seem* to have different assertability conditions.

Here is the step-by-step reconstruction of the objection.

1. Let *c* be a conversational context in which it is common belief that liquorice fails to taste good to one of the addressees. The multicentered worlds content expressed by an assertion of ‘Liquorice is tasty’ in *c* = the multicentered worlds content expressed by an assertion of ‘Liquorice is tasty to us’ in *c* = {$\left\langle w,\;t,\;\langle x_{1},\;\ldots \rangle \right\rangle$: liquorice tastes good to $\left\langle x_{1},\;\ldots \right\rangle$ in *w* at *t*} [Premise]
2. For any *c'*, if two assertions made in *c'* express the same content (have the same truth-conditions), then they have the same assertability conditions in *c'*. [Premise]
3. So in *c*, an assertion of ‘Liquorice is tasty’ and an assertion of ‘Liquorice is tasty to us’ have the same assertability conditions. [from 1, 2]
4. But the two assertions do not have the same assertability conditions in *c*. The assertion of ‘Liquorice is tasty to us’ is infelicitous and the assertion of ‘Liquorice is tasty’ is felicitous. [Premise]
5. Contradiction [3, 4]
6. Hence premise 1 is false: the content expressed by an assertion of ‘Liquorice is tasty’ in *c* ≠ the content expressed by an assertion of ‘Liquorice is tasty to us’ in *c*, *pace* the predictions of the multicentered worlds model. [from 1, 5]

The objection crucially relies on the claim about the multicentered worlds model in premise 1 and the principle linking truth conditions and assertability conditions in 2. But notice that the conversational context *c* is not sufficiently specified to guarantee the truth of premise 1. ‘Tasty PRO_C_’ and ‘tasty to us’ express the same content only on one of several possible readings of ‘us.’ The first-person plural pronoun ‘we’/‘us’ can pick out any group that includes the speaker. In particular, it can pick out groups including none of the addressees, some or all of the addressees.^54^ It is only in contexts in which ‘us’ picks out the group consisting of speaker, all addressees, and no one else that ‘tasty PRO_C_’ and ‘tasty to us’ express the same content in conversation. So the context *c* has to be a context that triggers this contextual interpretation if it is to establish the truth of premise 1. But this use of ‘tasty to us’ seems rare. Typically, PPTs are explicitly relativized to present people to mark a difference between them; hence the use of ‘tasty to me’ when retreating from a bare taste claim in the face of opposition. Likewise, the more natural use of ‘tasty to us’ is the exclusive reading, which underlines that some food tastes good to some group including the speaker, even if not to (all of) the addressees. So the scope of cases of which premise 1 is true is limited.^55^

The reason the objection fails, however, is that premise 4 is false. The assertions of ‘Liquorice is tasty’ and ‘Liquorice is tasty to us’ do have the same assertability conditions in a suitable context *c* in which ‘us’ picks out the conversational group. Both assertions are not appropriately assertable in *c* according to the strong group-centric norm because it is not true that liquorice tastes good to all of the conversational participants. But there may very well be good reason to think that agreement is still possible because the disagreeing addressee is in a position to accommodate. So both assertions are appropriately assertable according to the weak speaker-oriented norm of assertion. Our impression that the assertions come apart in appropriate assertability is due to the fact that the difference in overt linguistic material makes different purposes and thus different norms of assertability salient. The speaker-oriented purpose of a bare taste assertion, even in a situation in which it is understood that someone in the conversation disagrees with the claim, is to voice the speaker's perspective and persuade the hearers to adopt that perspective. As long as there is a chance that hearers can be persuaded, the assertion satisfies the weak norm. Our judgments of appropriate assertability of ‘Liquorice is tasty’ are guided by the speaker-oriented weak norm. In contrast, the explicit relativization ‘to us’ in ‘Liquorice is tasty to us’ makes the group's perspective on the tastiness of liquorice salient and draws attention to the purpose of maximal cooperativeness, which is geared at getting the group's perspective right. In *c*, where it is understood that the perspectives of the members diverge, ‘to us’ makes salient that no joint perspective is in place. Thus ‘to us’ draws attention to the falsity of the assertion's content and the failure of the strong norm. As a result, the speaker-oriented purpose gets trumped in salience, and our judgments are guided by the strong group-centric norm.^56^

## 12. Conclusion

I have argued that the conflict between a Lewisian view of belief as self-location and the received Lockean picture of communication can be resolved by conceiving of the contents of mental attitudes and speech acts as sets of multicentered worlds – possible worlds ‘centered’ on a sequence of individuals at a time. Multicentered worlds content is the kind of centered information that is transferred from speaker's head to hearer's head in successful communication. Communication, on the multicentered worlds view, is the project of distinguishing between possible ways the group of interlocutors might be and involves the coordination of participants' individual perspectives. The point of assertions about matters of taste is to reach a joint perspective. The point of *de se* assertions is to establish the speaker's individual possibilities.^57^

## Footnotes

1 See for instance, Castaneda (1966, 1967), Perry (1977, 1979), and Lewis (1979a, 1983a)

2 See for instance, Egan (2005, 2007, 2010a), Lasersohn (2005) and Stephenson (2007a)

3 *De se* attitudes are often understood in a wider sense to include *de nunc* attitudes – thoughts about when one is such as the thought *that the meeting starts now* – and thoughts about one's current location in space such as the thought *that this is the Mt. Tallac trail*. For simplicity, I will here focus on the narrow class of *de se* attitudes that are characteristically expressed by using 1st-personal pronouns.

4 The view on which centered worlds *are* triples of a world, time, and persisting individual is attributed to Lewis (1979a, 1983b). The term *centered world* was originally coined by Quine (1969). For Quine, centered worlds are spacetime regions of worlds – quadruples ⟨w,x,y,z,t⟩ consisting of a world *w*, a location in space given by coordinates *x*, *y*, and *z*, and a time *t*. As Liao (2012) points out, both the Quinean conception and the Lewisian conception of centered worlds above face counterexamples: intuitively different ways one might be that, on the respective accounts, are identified with the same set-theoretic object. For instance, if I travel back in time to the year 2000, ⟨@, DK, 2000⟩ picks out both my younger and my time-traveling older self. If a conscious ghost is co-located in my current spacetime region, the same quadruple ⟨w,x,y,z,t⟩ picks out the ghost and me. One way to accommodate such cases is to take possible individuals as primitives, individuated by their unique ‘identity’ properties. (My current and my older self are different possible individuals, and so am I and a co-locating ghost.) Another option is to take centered worlds as primitives – positions for individuals to occupy within worlds – rather than identify them with some set-theoretic object (Egan 2009, 253 n.3). For my purposes here, it will be safe to put aside time-traveling and co-location cases and remain neutral with respect to the nature of centered worlds. For convenience of exposition, I will assume that a ⟨w,t,x⟩-triple represents a centered world.

5 A second kind of argument are ignorance arguments, which purport to show that while one may know all relevant standard (possible worlds) propositions, one may still lack knowledge about oneself (see for instance Lewis's (1979a) two gods case, the dungeon case in his (1983a), and Perry's (1979) case of Lingens in Stanford Library). A third motivation for centered worlds content comes from the semantics of attitude verbs like ‘expect, want ’, and ‘imagine ’, whose truth conditions have been argued to be sensitive to the ascription of *de se* attitudes (see e.g. Morgan 1970; Chierchia 1989).

6 Cf. Lewis (1979a, 525–526). Perry (1977, 1979) draw the lesson that Hume and Heimson believe different propositions and that their doxastic similarity is to be accounted for by their shared ‘belief state’ – roughly, the first-personal mode of presentation of the believed propositions. For reasons to prefer Lewis' account over Perry's see Lewis (1979a, § 12).

7 A property determines its extension at possible worlds and times. Given a world and time, it determines the set of individuals that instantiate the property at that world and time. A property can thus be thought of as a function from worlds and times to sets of individuals. This function is equivalent to a function from world-time-individual triples to truth values. The set characterized by the latter function is a set of world-time-individual triples ⟨w,t,x⟩ such that *x* instantiates the property at *t* in *w* (cf. Lewis (1979a, 532)).

8 It is worth mentioning intermediate positions, as in Chierchia's (1989) work on the semantics of attitude ascriptions, according to which a property is ascribed in the case of *de se* attitudes but a standard proposition is ascribed in the case of non-*de se* attitudes.

9 See Nolan (2006), however, for objections to the claim that one can do with centered worlds whatever one can do with possible worlds.

10 Further arguments for the interestingness of taste contents, advanced by relativists, include arguments from retraction (e.g. MacFarlane 2014) and eavesdropping (e.g. Egan 2005).

11 Standard relativist arguments from disagreement target their main rival, simple contextualism. Objectivism, according to which tastiness is a monadic property of objects, can explain disagreement in terms of contradictory contents. On the objectivist view, Ben asserts the boring content that the cookie is (objectively) tasty, which Anna denies by asserting its negation. Some relativists have made the stronger argument that disputes about taste give rise to *faultless* disagreement – ‘where one person asserts or believes some sentence content, and another person believes or asserts its negation, but where neither person has made a mistake ’ (Lasersohn 2009, 360). Objectivists struggle with an explanation of the faultlessness of both Ben's and Anna's assertions. The literature on disputes between relativists, contextualists, objectivists and expressivists has grown considerably in recent years. For a beautifully clear, early synopsis of the available options, see Lasersohn (2005, sections 3–4). For a recent overview, see MacFarlane (2014).

12 Egocentricity also applies to belief in boring centered contents, even though intuitively, the appropriateness of one's belief that, say, snow is white depends on more than just one's own location. However, an agent is correctly located by a boring centered content if and only if she and all of her worldmates are correctly located by it. Thus it is only the appropriateness of believing interesting centered contents that depends *merely* on the location of the attitude holder.

13 For the attribution of the ‘Lockean’ picture of communication to Locke and for a defence of the picture against criticism, see Pagin (2008).

14 A complete account of linguistic communication will have to account for pragmatically conveyed information, disambiguation, indirect speech acts and other pragmatic phenomena as well. Here, I am interested only in that part which accounts for the connection between mental content, linguistically expressed content, and compositional semantic theory. By a ‘theory of communication’, I shall mean, following Lewis (1975, 1980), a systematic restatement of speakers' common knowledge of their practice of linguistic communication.

15 See Torre (2010) for reasons to think that communication of *de se* information is possible.

16 This problem was first raised by Stalnaker (1981, 146–147).

17 There are alternative responses to the conflict. They fall in two groups: Those which give up the self-locating account of mental attitudes and preserve the Lockean picture of communication; and those which hold on to the self-locating account of mental attitudes but abandon the Lockean picture of communication. For the *de se* problem, the first group of responses is represented by Perry (1977, 1979) and Stalnaker (1981), and the second group by Egan (2007, 2010a), Kölbel (2013), Moss (2012), Heim (2004), Weber (2013), and Moltmann (2010). With the exception of Egan and Moltmann, none of these views address the incompatibility problem. See Kindermann (2016) for a comparison of these views of *de se* communication. Kindermann (2012) sketches ways of extending these views to cover taste attitudes and discusses possible ways in which they might address the incompatibility problem. Let me here just state that even those of the extended alternatives that provide a solution to both the *de se* and the incompatibility problem still require giving up one of the natural and attractive views that the multicentered worlds account reconciles.

18 ‘Multicentered world’ seems to be the widely used term in the literature now. While multicentered/sequenced worlds do not uniformly play the same roles in theories that make use of them (cf., e.g. Ninan 2012; Stalnaker 2014) the terminological lines between ‘sequenced world’ and ‘multicentered world’ do not track such differences. For uses of ‘sequenced world ’, see Ninan (2010b) and Kindermann (2012).

19 Lewis (1983a, 28) himself provides the idea of worlds with multiple centers but does not use them to account for centered communication: ‘Besides possible individuals, world-sized and smaller, there are still other possibilities: joint possibilities for two or more individuals. These are ordered pairs, triples, etc. … or even infinite sequences of possible individuals, all from the same world. An ordered pair of compossible individuals, for instance, is a way that a pair of individuals might possibly be.’

20 The model I develop here differs from Ninan's (2010b) and Torre's (2010) accounts in a number of technical and substantial ways. Most importantly, neither Ninan's nor Torre's account are attempts to preserve the Lockean picture. While both take speech act content to be multicentered worlds content, on Torre's account mental content is centered worlds content (Torre 2010, 112). For Ninan (2010b, 15), the account of belief in multicentered worlds content is explained in terms of belief in centered worlds content, which suggests that centered worlds belief is taken to be the fundamental notion of belief. Moreover, the present model differs in its definition of speech act content (Section 8) from Ninan's account, which faces a problem once the required notion of *inverse* is generalized for sequences of more than two interlocutors.

21 The set of conversational contexts is a proper subset of the set of multicentered worlds – those multicentered worlds in which the individuals of the sequence are in a conversation with each other. For a given moment in a conversation, there are as many formal objects I call conversational contexts as there are ways of combining the participants into an ordered sequence.

22 Pearson (2016) makes use of an interesting and slightly different (Lewis-based) notion of group location in her semantics of partial control predicates such as ‘expect’ in sentences such as ‘John expected to go on vacation together’ (her example). The latter sentence says, roughly, that Johns expected of a group that includes himself to go on vacation together. So Pearson's *de se* analysis of partial control predicates also involves location of a group in some sense. Since her semantics formally employs orthodox Lewisian centered-worlds content, we could say that her account of partial control predicates such as ‘expect’ has the subject *locate herself in a group* which has a certain property (such as *going on vacation together*).

23 Cf. Section 8 on conversational contexts and conversational roles

24 In solitary thinking and soliloquy, in which the thinker is not addressing anyone in a second-personal way, we can think of belief as location of the group that consists just of the thinker herself. That is, her belief content is a set of multicentered worlds whose sequences have a single center – i.e. it is a centered worlds content. So in solitary contexts, belief naturally amounts to self-location. n-Belief in a conversational context captures self-location if we allow the identity relation that a thinker bears to herself as a limit case of a conversation-establishing relation in clause (ii).

25 Ninan (2010b) and Torre (2010) endorse something like conservative multicentering for *de se* communication.

26 Note that the Lockean picture does not require that interlocutors believe the same content in the same way. Thus the multicentered worlds view preserves the Lockean picture even when the speaker may *m*-believe it and the hearer may *n*-believe it.

27 For more discussion of conservative and radical multicentering, see Kindermann (2016). The account of n-Belief in a conversational context bears similarity to the account of attitudes with multicentered worlds content in Ninan (2012). Aside from technical aspects, the two accounts differ in the following respects. First, a multicentered world in Ninan (2012) represents a single agent's perspective on the world, whereas a multicentered world on the present view represents a group's perspective on the world. Second, an agent's overall belief state, including all cognitively relevant aspects, can be modeled by a set of multicentered worlds in Ninan (2012) , whereas a set of multicentered worlds needs to be supplemented with a specification of the agent's belief relation (1-belief, 2-belief, *n*-belief) on the present view to capture all aspects of cognitive significance. Third, multicentered worlds belief on the present view allows for the Lockean communication of *de se* attitudes (a single content is the object of speaker's and hearer's beliefs). In contrast, multicentered worlds content of *de se* attitudes in Ninan (2012) are incommunicable (cf. 2012, 38 n. 25). Fourth, the multicentered worlds account in Ninan (2012) is designed to solve a problem with counterfactual *de re* attitudes (such as imagining and desiring) which Lewis' (1979a) centered words account faces. The present account of n-Belief in a conversational context cannot be extended in a straightforward way to counterfactual attitudes without running into this problem.

28 For instance, a semantics with multicentered worlds indices can also be formulated by modifying Lasersohn's (2005, 2008) semantics for predicates of personal taste. Pronouns could alternatively be treated as variables and receive their semantic value from an assignment function (cf. Heim 1994; Heim and Kratzer (1998, ch. 9), von Stechow 2004), if appropriate changes were made to the definition of speech act content in Section 8.

29 For a clear exposition of an ‘orthodox’ version of the system with possible worlds as the only coordinates of the index, see Heim and Kratzer (1998, ch.12) and von Fintel and Heim (2011). For Kaplan's original system, see Kaplan (1989).

30 The index may or may not be a multicentered world depending on whether a possible situation corresponds to the combination of world, time, and individuals. For instance, ⟨@, 6 May 1902, ⟨Frege, Russell⟩⟩ is a multicentered world but ⟨@, 6 May 2019, ⟨Frege, Russell⟩⟩ is not, since Frege and Russell inhabited @ in 1902 but not in 2019. Both triples, however, can be values of the index.

31 The subscript ‘*e*’ indicates *y*'s semantic type. *e* is the semantic type of individuals, *s* stands for worlds, *i* for times, and *t* for truth values; combinations such as ‘*et*’/⟨e,t⟩ stand for functions from the first type (*e*) to the second (*t*). Read ‘[*λ*y${}_{e}$. y is hungry in *w* at *t*]’ as ‘the function which maps every y${}_{e}$ to 1 (truth) if y is hungry in *w* at *t*, and to 0 (falsity) otherwise.’

32 I shall for the most part restrict my attention to paradigm examples of predicates of personal taste such as ‘fun’ and ‘tasty’. The grammatical demarcation of a class of predicates of personal taste from aesthetic and other evaluative predicates is difficult, as Lasersohn (2005) observes (see Lasersohn (2008, §1.2), though, for a non-definitional demarcation of predicates of personal taste based on the interaction of perspective with scalarity)

33 First-personal uses of PPTs must be distinguished from so-called exocentric uses, in which the taste preferences of a contextually salient individual are at issue, as well as from explicitly relativized uses with overt prepositional phrases such as ‘for Ben.’ In this short introduction, I focus on first-personal uses, but see footnote 35 on exocentric and explicitly relativized uses of PPTs.

34 Treating PPTs as two-place predicates provides a straightforward handling of uses of PPTs with an overt prepositional phrases such as ‘for Ben’ in ‘The roller coaster is fun for Ben’ and of so-called exocentric uses of PPTs, which have no overt experiencer but in which the context of utterance makes a particular individual salient, as in (1) (adapted from Lasersohn (2005, 672)):

**Table:**

(1)	Anna:	How does Bill like the rides?
	Ben:	Well, the merry-go-round is fun, but the water slide is a little too scary. The treatment of these two uses of PPTs on the multicentered worlds semantics does not differ from Stephenson's treatment. The interested reader is referred to Stephenson (2007a, §4.4).

35 A note on negation: a negated sentence like ‘This cookie is not tasty PRO_C_’ says, roughly, that for each of the interlocutors $\left\langle x_{1},\;\ldots ,\;x_{n}\right\rangle$, the cookie does not taste good to them. Another reading, on which the sentence says that it is not the case that the cookie tastes good to each and all interlocutors, does not seem to be available (Note the oddity of responding ‘That's right’ despite one's liking the taste of the cookie, simply on the evidence that there is one person among the interlocutors, the speaker, who doesn't seem to like the cookie's taste.) This is what we should expect given that PRO_C_ is a plural expression whose denotation is a plurality of (singular) individuals. ‘Is tasty’ behaves like many predicates in combining with plural expressions to yield distributive readings: if the predicate can be truly applied to a plurality, it can also be truly applied to each of its members. Many theorists posit a covert distributivity operator, which optionally attaches to the VP to yield distributive readings (see e.g. Roberts 1990; Lasersohn 1998; Nickel 2012 for an overview). The distributivity operator can be inside the scope of negation, yielding the first reading: *It's not the case that each of $\left\langle x_{1},\;\ldots ,\;x_{n}\right\rangle$ is such that the cookie tastes good to them*. This reading is true even when the cookie tastes good to many of them. In this scenario, the second reading in which the distributivity operator takes wide scope is false: *Each of $\left\langle x_{1},\;\ldots ,\;x_{n}\right\rangle$ is such that the cookie does not taste good to them.* The unavailability of the first, narrow-scope reading is due to a presupposition of homogeneity: A (definite) plural expression presupposes that each individual in its denotation behaves the same with regard to the predicate with which it combines (see Schwarzschild 1993; Löbner 2000). In our case, the presupposition of homogeneity says that the predicate ‘λx . the cookie is tasty to *x*’ applies either to all of $\left\langle x_{1},\;\ldots ,\;x_{n}\right\rangle$ or to none. This rules out the narrow-scope reading on which the negated sentence is true if the cookie fails to be tasty to some but not all of $\left\langle x_{1},\;\ldots ,\;x_{n}\right\rangle$. PRO_C_ is thus similar to other plural expressions. Sentences that take an overt plural experiencer argument, such as ‘This liquorice is not tasty to the children ’, also have only the wide-scope reading, according to which each of the children is such that the liquorice does not taste good to them. Note the oddity of (i), which would require a narrow-scope reading to be true.

(i) [Context: Lucy and Trevor are among the four children being talked about.]
  ? This liquorice isn't tasty to the children, though it is tasty to Lucy and Trevor.

36 It bears mentioning that the semantics for PPTs in this section, in connection with the multicentered worlds view, is compatible with both a relativist and a contextualist outlook on PPTs. More precisely, as I show in Kindermann (2012), the multicentered worlds view with the above semantics allows of refinements that service either truth relativists or nonindexical contextualists (to use MacFarlane's (2009, 2014) classification of views). The decision in favor of or against the multicentered worlds view is thus largely independent of one's views on relativism vs contextualism. The decision between relativism and contextualism turns primarily on empirical data from speakers' use of PPTs, such as eavesdropping, retraction, and disagreement.

38 Ninan (2010a) offers a clear and illuminating exposition of Lewis' point, as well as the various options for defining speech act content in a Kaplan-style intensional semantics. See also Rabern (2012), Stanley (1997a, 1997b). Dummett also distinguished between ‘assertoric content’ (≈speech act content) and ‘ingredient sense’ (≈compositional semantic value) (Dummett 1973, 1991).

39 The Kaplan diagonal abstracts over entire contexts. It thus affects the interpretation of all expressions whose semantic value varies with context. For instance, the speech act content of an assertion of ‘This is Big Ben’ in a conversation with two interlocutors is something like (i):

(i) {$\left\langle w,\;t,\;\langle x_{1},\;x_{2}\rangle \right\rangle$: the object demonstrated in $\left\langle w,\;t,\;\langle x_{1},\;x_{2}\rangle \right\rangle$ is Big Ben in *w* at *t*}

Treating the contribution of all context-sensitive expressions on a par with that of 1st- and 2nd-personal pronouns is required if we accept that the class of *de se* attitudes include *de hic* (locational) attitudes and *de nunc* (temporal) attitudes, whose expression involves locationally and temporally context-sensitive vocabulary. (Perry's cases of the hiker lost in the wilderness and of the professor desiring to attend the department meeting on time provide motivation for widening the class of *de se* attitudes (Perry 1979, 4); cf. fn. 3). It is worth noting that we could adopt a semantics of pronouns and other context-sensitive expressions and diagonalize in a way that allows ‘this’ and similar expressions to provide their referents to speech act content.

40 Multicentered worlds speech act content depends on facts about the context, namely the number of participants in the conversation. As a result, assertions of the same sentence by the same speaker may express different sets of multicentered worlds in contexts that differ only with respect to the number of interlocutors. There is a clear sense, however, in which ‘what is said’ by these assertions is the same. A multicentered worlds content is a way of distinguishing between alternative possibilities – between alternative ways a number of people might be. It can be understood as a partition of a space of possibilities, a space which may itself differ in kind from context to context, depending on the number of people whose possibilities are represented. The same content, understood as a partition, can thus yield different sets of multicentered worlds – sets of worlds with sequences of different length – depending on the space of possibilities which it partitions.

41 Stalnaker's views on the pragmatics of communication are developed in his 1970, 1974, 1978, and 2002, among others.

42 In the default case, the common ground will be common knowledge or common belief. But it need not be. Interlocutors may take non-public attitudes towards what is presupposed, depending on the purpose of the conversation. When the purpose is to establish truth, the attitude is, plausibly, knowledge or belief; when speakers are interested in exploring a hypothetical situation, the mutually recognized non-public attitude is supposition; asf. What the right non-public attitude to take is may itself be a matter of negotiation between interlocutors. I will for the most part focus on the default case where the common ground is common belief. Even when presupposition entails belief, however, the converse is not true. Given the public nature of presupposition, a speaker typically believes a variety of things she does not believe her audience to believe, or she may believe that her audience believes them but not that they believe that she believes them, etc.

43 Stephenson (2007a, 2007b) develops a view of the Stalnakerian common ground on which the conversation's context set is a set of centered worlds. Her approach shares with the present proposal that the center represents the group of conversational participants: ‘I propose that for all the triples in the context set for a conversation, the judge [individual] element represents the plurality of the group of participants in the conversation’ (2007a, 509). Moreover, Stephenson's view preserves the Lockean picture of communication. However, her view can do without additional structure for the group-element (not a sequence but a single individual) because it is an account of taste attitudes in communication only. The conflict in Section 4 showed that adding interesting centered contents for the communication of *de se* attitudes would have the counter-intuitive consequence that talk about oneself would lead hearers to update with attitudes *about themselves* rather than the speaker. So Stephenson's view doesn't present a solution to the conflict arising from interesting centered worlds content in the communication of both *de se* and taste attitudes.

44 Note that to assess whether {$\left\langle w,\;t,\;\langle x_{1},\;x_{2}\rangle \right\rangle$: $x_{2}$ is mad in *w* at *t*} is true from $\langle w_{c},\;t_{c},\;\langle$Ben, Heimson⟩⟩, one need not be able to identify $x_{2}$ *de re* as Heimson. We use the names ‘Ben’ and ‘Heimson’ to state what the conversational sequence is merely for convenience. All that is needed to determine whether some content is true from the conversation's perspective is the ability to keep participants apart and consistently track them in derivations of content from context. For participants in one-to-one conversations, the ability to distinguish between oneself and not-oneself and to track them, respectively, suffices.

45 By ‘bare taste assertions’ I mean first-personal uses of PPTs that are covertly relativized to PRO_C_ (cf. Section 7). Other uses of PPTs are attested in the literature, e.g. uses on which they are covertly relativized to a contextually salient experiencer (‘exocentric uses’, cf. fn. 35) or on which an experiencer variable is bound by a quantifier (see e.g. Schaffer 2011). In this section, I put such uses aside.

46 Ben's assertion in (21c) amounts to a ‘partial retraction’ of his first assertion. Cf. Pearson (2013, §4.6) on ‘partial retraction’ and what (Schaffer 2011) calls ‘entrenchment’ for a related phenomenon. The example in (21) is adapted from Pearson (2013, § 4.6).

47 Of course, an explanation of the conversational impropriety of (22c) may appeal to other norms than the norm of assertion. But the example shows at the very least that egocentric truth norms are blind to conversational circumstances that transcend the speaker's individual perspective.

48 The negation, ¬Φ, of a bare taste sentence Φ on a first-personal use has two readings depending on the scope of the negation. Where Φ is the sentence ‘*X* is tasty ’, the default wide-scope reading says, very roughly: it is not the case that *X* is tasty to all of the conversational participants. The default narrow scope reading says: *X* is not tasty to either of the conversational participants. For the definition of Joint perspective on p to deliver the intuitively correct result, ¬p must be the content expressed by the narrow scope reading of ¬Φ. Thus, where Φ is a bare taste sentence, ⌜¬p⌝ does not denote the complement set of *p*. On negated taste claims, see also footnote 36 above.

49 See Egan (2007), Egan (2010a) and López de Sa (2008) for two accounts on which a presupposition of relevant similarity is a necessary condition for the felicity of assertions of bare taste sentences.

50 See for instance, Stevenson (1963, ch.2)

51 It is widely (though not uncontroversially) assumed that a rule of accommodation applies to speech acts that carry presuppositions: If a speech act requires presupposition *q* to be appropriate and *q* is not yet presupposed, then *ceteris paribus* the presupposition *q* comes into existence (cf. Lewis (1979b, 340)).

52 Relativists like Egan (2010a, 273), MacFarlane (2007, 20), and Recanati (2007, 93 n.35) have observed the link between the process of accommodation (in Lewis' sense) and the purpose of establishing a shared viewpoint. For Richard (2008, 101), the process of ‘accommodation and negotiation’ can be found wherever we use vague scalar expressions that give rise to relative truth.

53 The discussion in this section owes much to Egan (2010a), with whose general outlook on the reasonableness of disputes about taste I am in large agreement. Of course, no claim is made about the converse agreement.

54 More precisely, ‘us’/‘we’ allows of inclusive and exclusive readings. On the exclusive reading, the referent is a group that includes the speaker but excludes the addressee (‘I and others but not you’). On the exclusive reading, ‘tasty’ and ‘tasty to us’ do not express the same content in conversation. On inclusive readings, the addressee is included in the group referred to by ‘we’/‘us.’ Inclusive readings can further be distinguished. On so-called 1+2 readings (the numbers refer to first and second person), the group referred to consists only of speaker and addressee(s). On 1+2+3 readings, the group referred to consists of speaker, addressee(s) and third parties.

55 It is also worth noting that premise 2 is far from obvious. Indeed it is routinely dismissed in accounts of the communicative effects of assertions that appeal to pragmatic implicatures, semantic or pragmatic presuppositions, or information structural properties like focus. In the example above, however, I do not think that any of these phenomena is responsible for the perceived difference in assertability conditions. So I will not dispute premise 2 here.

56 Recanati (2008) endorses what he calls ‘moderate relativism’ about aesthetic predicates on which ‘It is beautiful’ means something like *It is beautiful for us*, that is, for the community to which the speaker and his audience belong.' (59) He discusses a problem similar to the objection above: Why, in light of opposition, do speakers sometimes not retreat to a weaker explicit statement about their own aesthetic perspective but keep asserting ‘It is beautiful’? Recanati suggests that the disputants appeal to a community standard which they try to shape with their assertions, with ‘one foot in the future’ (quote from an unpublished manuscript by Johan Brännmark that Recanati cites). What Recanati's account does not explain is why it is significantly less natural to try and shape one's community's standards by making the explicitly relativized aesthetic assertion with the same content. It is an advantage of the pragmatic account I favor that it can account for the difference between bare uses of PPTs and uses on which the predicate is explicitly relativized to the conversational group.

57 I am grateful to many people for their advice, support, comments and criticism: to Andy Egan, Patrick Greenough, Torfinn Huvenes, Dilip Ninan, Jonathan Schaffer, and Brian Weatherson for numerous discussions and substantive help; to Derek Ball, Michael Caie, Josh Dever, John MacFarlane, Hazel Pearson, Simon Prosser, Tamina Stephenson, Stephan Torre and Seth Yalcin for very helpful comments and suggestions; and to an anonymous referee for this journal for constructive comments. Parts of this paper were presented at the Wolheim Society at UC Berkeley, the ‘Meaning, Context and Implicit Content’ conference in Cerisy, SPE4 in Bochum, the Logos workshop ‘Semantic Content and Context Dependence’ in Barcelona, and at Arché's ‘Contextualism and Relativism Workshop II’ in St Andrews. Thanks to the audiences at these occasions.
